# Association between blood inflammatory status and the survival of tuberculosis: a five-year cohort study

**DOI:** 10.3389/fimmu.2025.1556857

**Published:** 2025-03-21

**Authors:** Yating Ji, Qingyao Xie, Wei Wei, Zhen Huang, Xuhui Liu, Qi Ye, Yanping Liu, Xiaoyu Lu, Yixiao Lu, Renjie Hou, Qingping Zhang, Yanzi Xu, Jianhui Yuan, Shuihua Lu, Chongguang Yang

**Affiliations:** ^1^ School of Public Health (Shenzhen), Shenzhen Key Laboratory of Pathogenic Microbes and Biosafety, Sun Yat-sen University, Shenzhen, Guangdong, China; ^2^ Department of Tuberculosis, Shenzhen Third People’s Hospital, Shenzhen, Guangdong, Guangdong, China; ^3^ National Clinical Research Center for Infectious Disease, Shenzhen, Guangdong, China; ^4^ Department of Biochemistry and Molecular Biology, Tulane University School of Medicine, New Orleans, LA, United States; ^5^ Department of Tuberculosis, Shanghai Public Health Clinical Center, Fudan University, Shanghai, China; ^6^ Infectious Disease Prevention and Control Department, Nanshan District Center for Disease Control and Prevention, Shenzhen, Guangdong, China; ^7^ School of Public Health (Shenzhen), Guangdong Provincial Highly Pathogenic Microorganism Science, Guangzhou, China

**Keywords:** blood inflammatory status, inflammation, tuberculosis, prognosis, survival analysis, cohort study

## Abstract

**Background:**

Blood inflammatory status is closely associated with tuberculosis (TB) progression. Emerging inflammatory indices from different leukocyte subtypes have become a prognostic hotspot for various diseases, yet their application in TB prognosis remains limited. This study aims to assess the impact of inflammatory status on TB patients’ prognosis and its potential as a prognostic indicator to optimize prognostic assessment and therapeutic strategies.

**Methods:**

This study included 4027 TB patients admitted to a tuberculosis-designated hospital in Shenzhen from January 2017 to December 2022. Patients were classified into three inflammatory statuses (Q1-Q3) based on each index’s level. We conducted Cox regression and restricted cubic splines (RCS) analyses to evaluate the association between inflammatory status and unfavorable outcome, subgroup analyses to understand heterogeneous associations among subpopulations, and receiver operating characteristic (ROC) analyses to evaluate the prognostic performance of inflammatory status on TB treatment outcomes.

**Results:**

During 48991.79 person-months of follow-up involving 4027 patients, 225 unfavorable outcomes occurred. Multivariable Cox regression indicated that the Q3 levels of CAR, CLR, dNLR, NLR, SII, and SIRI increased the risk of unfavorable outcome by 45%-99% (HR: 1.45-1.99, all *P*<0.050), whereas ENR reduced the risk by 29% (HR: 0.71, *P*=0.040) compared to Q1. RCS curves revealed linear associations with unfavorable outcome that were positive for CAR, CLR, dNLR, SII, and SIRI, negative for ENR (all *P* for nonlinear>0.050), and nonlinear for MLR, NLR, and PNI (all *P* for nonlinear<0.050). Subgroup analyses identified heterogeneous associations across age, sex, BMI, comorbidities, and drug resistance (all *P* for interaction<0.050), with attenuated risk effects of CAR, CLR, dNLR, and SII in patients aged 30-60 years, male, BMI≥24.0 kg/m², smokers, retreatment cases, and those with tumor. ROC analysis demonstrated stable predictive performances of inflammatory status (AUC: 0.785–0.804 at 6-month, 0.781–0.793 at 9-month, and 0.762–0.773 at 12-month), and the combination of the inflammatory status significantly optimized the prognostic performance of the basic model (9-month AUC: 0.811 vs 0.780, *P*=0.024; 12-month AUC: 0.794 vs 0.758, *P*=0.013).

**Conclusion:**

Pretreatment blood inflammatory status effectively predicts the treatment outcome of TB patients. Our findings hold significant clinical value for TB patient management and warrant prospective evaluation in future studies.

## 1 Introduction

Tuberculosis (TB) remains a significant global health challenge, once again recognized by the WHO as the leading cause of mortality among infectious diseases (1). Ongoing research efforts are focusing on identifying indices and risk factors to assess disease status, predicting TB prognosis, and ultimately improving personalized treatment and reducing mortality (2–5). Inflammation plays a critical role in the onset and progression of TB. While pro-inflammatory mechanisms aim to control pathogens, excessive and prolonged responses may result in granuloma expansion and tissue damage (6), thereby promoting TB development and progression (7). However, most studies emphasize clinical factors, including age, sex, weight, homelessness, drug use, diabetes, and HIV (8–10), yet largely neglect inflammatory indices. Key immune-related cells in peripheral blood serve as reliable indicators of chronic inflammation (11). Given the limitations of single-cell counts in capturing systemic inflammation, novel inflammatory indices based on diverse leukocyte subtypes have become a focal point (11).

To evaluate the impact of inflammation on tuberculosis treatment, recent studies have incorporated inflammatory indices into TB prognostic prediction models. Ciccacci et al. (12) demonstrated that combining C-reactive protein (CRP) with malnutrition predicted short-term mortality in HIV-positive TB patients. Cynthia et al. (13) found that CRP>100mg/L and MLR are effective predictors of mortality in elderly TB patients. Qi et al. (14) reported that six inflammatory indices, including neutrophil-to-lymphocyte ratio (NLR), platelet-to-lymphocyte ratio (PLR), and monocyte-to-lymphocyte ratio (MLR), were elevated as prognostic risk factors for rifampicin-resistant/multidrug-resistant tuberculosis (RR/MDR-TB) patients. The combination of these six indices predicted mortality with an AUC of 0.823 (95%*CI*: 0.769-0.876). However, these studies focused exclusively on specific TB populations, which may limit their applicability in broader clinical practice. Additionally, fitting inflammatory indices to prognostic risk as a simple linear relationship may be inaccurate, as in some diseases a nonlinear relationship has been observed (15, 16). Meanwhile, systemic immune-inflammatory index (SII), systemic inflammatory response index (SIRI), and nutritional prognostic index (PNI) are regarded as comprehensive indices that more accurately reflect local immune responses and systemic inflammation (15, 17). However, their impact on TB treatment has not yet been fully evaluated. Here, we aimed to assess the impact of blood inflammatory status on TB prognosis and evaluate its potential as a prognostic indicator to optimize the prognostic assessments and treatment strategies.

## 2 Methods

### 2.1 Study setting and participants

**Figure 1:**
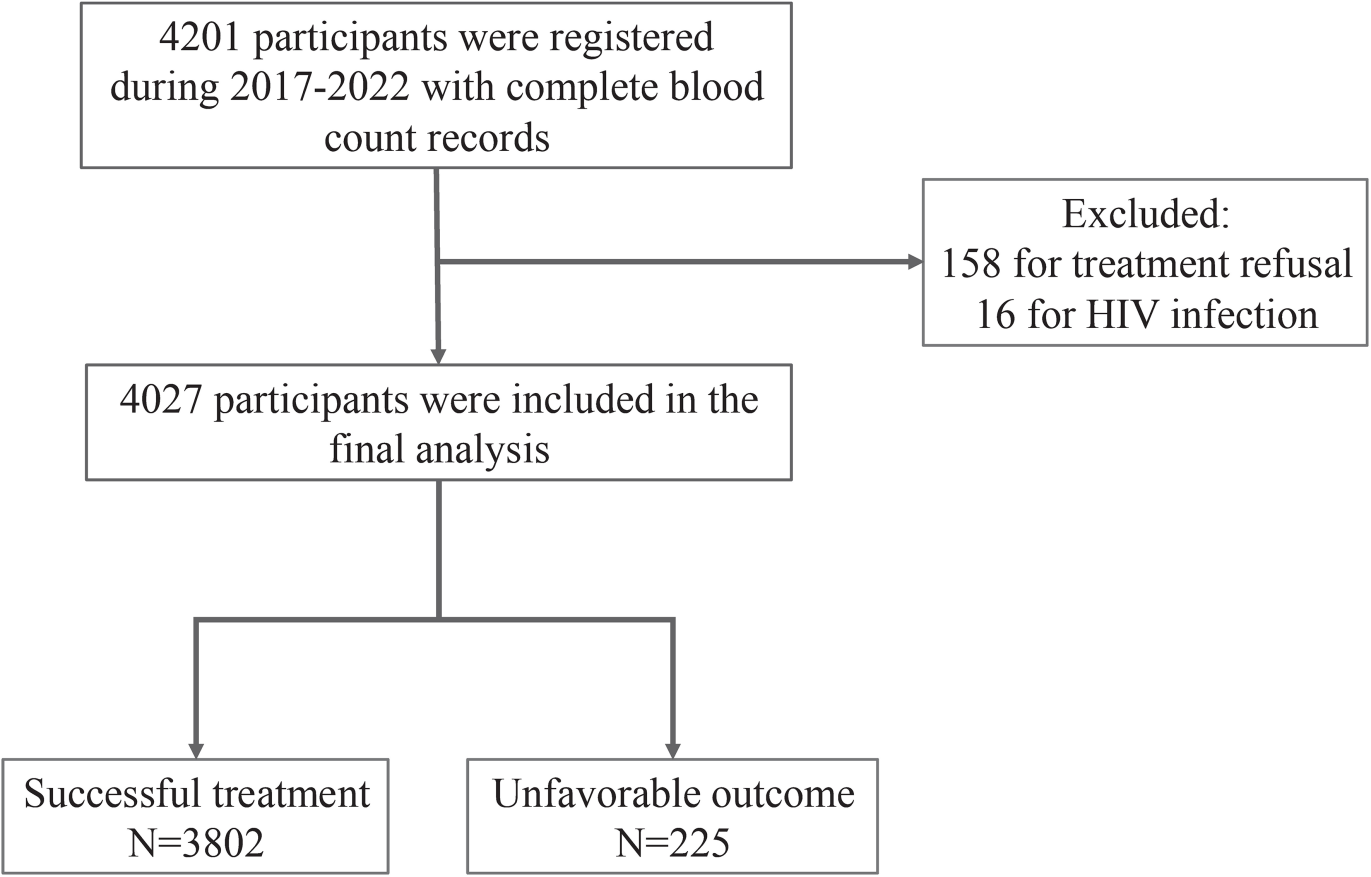
Flowchart of the eligible participants selection.

The study was conducted at the Shenzhen Third People’s Hospital, a major TB-designated hospital in Shenzhen, China. A total of 4201 pulmonary TB (PTB) patients with complete blood count records, with or without extrapulmonary TB (EPTB), were registered from January 2017 to December 2022. According to the national standard of diagnosis for PTB (18), confirmed PTB cases include individuals who are sputum smear microscopy positive, culture positive, molecular test positive or individuals who have pulmonary lesions of tuberculosis that have been confirmed by pathological examination (lung biopsy). Those who fail to meet the criteria for confirmed PTB are clinically diagnosed if other pulmonary diseases are excluded or their chest radiograph supports active PTB and they have any of the below: 1. PTB signs like cough, expectoration, hemoptysis; 2. Immunology evidence like strong purified protein derivative (PPD) skin test reaction, positive Interferon-gamma release assay (IGRA), positive MTB anti-body test; or 3. EPTB confirmed by pathological examination. Of these, 174 patients were excluded (**Figure 1**) due to treatment refusal (n=158) and HIV infection (n=16).

### 2.2 Data collection and definition

#### 2.2.1 Blood inflammatory indices

Blood test results collected from the laboratory information system at the start of treatment were used to calculate the following blood inflammatory indices (14, 19, 20): C-reactive protein to albumin ratio (CAR), C-reactive protein to lymphocyte ratio (CLR), derived neutrophil-to-lymphocyte ratio (dNLR), eosinophil-to-lymphocyte ratio (ELR), eosinophil to monocyte ratio (EMR), eosinophil to neutrophil ratio (ENR), monocyte to lymphocyte ratio (MLR), neutrophil to lymphocyte ratio (NLR), platelet to lymphocyte ratio (PLR), Prognostic Nutritional Index (PNI), Systemic Immune Inflammatory Index (SII), and Systemic Inflammatory Response Index (SIRI).

#### 2.2.2 TB treatment outcome definitions

According to the WHO guidelines, TB treatment outcomes are defined as follows: (a) Successful treatment, the sum of cured and treatment completed (21), and (b) Unfavorable outcome (22, 23), including treatment failure and died. Detailed definitions are shown in **Supplementary Table 1**. The survival time was defined as the interval between treatment initiation and the occurrence of any of the above-defined outcomes.

#### 2.2.3 Demographic and clinical characteristics

Demographic and other clinical characteristics of patients were prospectively collected form electronic medical record. Demographic information included age, sex, body mass index (BMI), domicile, employment status, alcohol consumption, and smoking status. Clinical characteristics included diagnostic delays (24) (patient-related, hospital-related, and total diagnostic delay), treatment category (initial and retreat), TB-related symptoms (cough, expectoration, fever, chest pain, hemoptysis, night sweats, fatigue, and weight loss), comorbidities (diabetes, hepatitis B virus, hypertension, tumor, anemia, and extrapulmonary tuberculosis), pathogenic status at baseline and after two months of treatment, cavity in pretreatment radiology imaging, and drug susceptibility test results (25, 26) [drug-susceptible TB (DS-TB), drug-resistant TB (DR-TB), multidrug-resistant TB (MDR-TB), rifampicin-resistant TB (RR-TB), rifampicin-susceptible while isoniazid-resistant TB (Hr-TB)]. TB-related symptoms, comorbidities, and cavity are binary variables with “yes” and “no” categories. The maximum percentage of missing covariate data was 29.60% (**Supplementary Table 2**). Similar to other clinical studies (27), we addressed the missing data using multiple imputations by chained equations under the assumption of missing at random, and pooled parameter estimates by Rubin’s rules.

### 2.3 Statistics analysis

Continuous variables with normal distributions were expressed as means ± standard deviations and compared by analysis of variance, while those with non-normal distributions were presented as medians with interquartile ranges and compared by the Wilcoxon rank sum test. Categorical variables were summarized as frequencies and percentages and compared by the chi-square test or Fisher’s exact test.

Patients were categorized into three statuses according to the 33rd percentile (P33) and 66th percentile (P66) of each blood inflammatory index (**Supplementary Table 3**): Q1 (<P33), Q2 (P33–P66), and Q3 (≥P66) (28). Kaplan-Meier estimates and Log-rank tests were employed to compare the cumulative incidence of unfavorable treatment outcomes across the different inflammatory statuses. Hazard ratio (HR) and 95% confidence interval (*CI*) were estimated in three Cox proportional hazards regression models: an unadjusted model (Model 1), an age, sex, and BMI adjusted model (Model 2), and a comprehensive adjustment for potential confounders (Model 3), encompassing work status, smoking, treatment category, expectoration, fatigue, weight loss, diabetes, hypertension, tumor, anemia, EPTB, cavity, drug susceptibility, and bacteriological result at the end of 2-month treatment, which showed significant associations with unfavorable outcome in univariate Cox regression (all *P*<0.050, **Supplementary Table 4**). A restricted cubic spline (RCS) analysis was conducted to explore potential nonlinear associations between blood inflammatory indices and unfavorable outcome. The number of knots in the RCS model were determined by the Akaike information criterion (AIC).

Subgroup analyses were conducted to evaluate the impact of inflammatory status on unfavorable outcome across different populations, stratified by age (<30, 30-60 and ≥60 years), BMI (<18.5, 18.5–24, and ≥24 kg/m²), sex, smoking status, comorbidities, cavity, and drug sensitivity. Additionally, receiver operating characteristic (ROC) curves were applied to assess the predictive value of blood inflammatory status on treatment outcomes (29). Two sensitivity analyses were performed to assess the robustness of the primary results following the approach of several clinical studies (22, 30). Sensitivity Analysis 1 utilized the multiple imputed data excluding patients with comorbidities. Sensitivity Analysis 2 was based on complete case analysis, excluding patients with missing data.

All analyses were conducted using R version 4.4.1 (R Foundation for Statistical Computing, Vienna, Austria). A *P*-value of <0.050 was considered statistically significant.

## 3 Results

### 3.1 Baseline information of the participants

Overall, 4,027 patients with PTB were included in this study (**Table 1**). The median age of the patients was 38 (IQR:27-55) years, with the majority being male (2504, 62.2%), migrant population (2978, 74.0%), and patients under initial treatment (3625, 90.0%). Cough (2961, 73.5%) and diabetes (752, 18.7%) were the most common TB-related symptom and comorbidity, respectively. At baseline, 3917 (97.4%) patients had bacteriological confirmation, and after two months of treatment, 280 (8.70%) remained positive. Cavity was observed in 766 (21.2%) patients on pulmonary radiological imaging. DS-TB comprised 2,445 cases (86.2%), followed by 162 MDR (5.71%), 134 RR-TB (4.7%), and 74 Hr-TB (2.6%) cases.

**Table 1:** The baseline information of the participants in this study.

Characteristics	Overall N=4027	Unfavorable outcome N=225	Successful treatment N=3802	*P*
Age/year	38 (27,55)	58.0 (42,73)	37.0 (27,53)	<0.001
Total Delay/day	31 (17,63)	32 (19,63)	31 (17,62)	0.242
Patient Delay/day	15 (6,38)	15 (6,34)	15 (6,38)	0.835
Hospital Delay/day	11 (7,16)	14 (6,20)	11 (7,16)	0.001
LOT/month	9.4 (7.0,12.0)	4.1 (2.3,7.2)	9.6 (7.2,12.0)	<0.001
Sex:				0.004
Female	1523 (37.8%)	64 (28.4%)	1459 (38.4%)	
Male	2504 (62.2%)	161 (71.6%)	2343 (61.6%)	
BMI/kg·m^-2^				0.001
<18.5	1032 (31.5%)	64 (40.0%)	968 (31.0%)	
18.5~24.0	1874 (57.1%)	69 (43.1%)	1805 (57.9%)	
≥24.0	374 (11.4%)	27 (16.9%)	347 (11.1%)	
Domicile:				0.357
Resident	1049 (26.0%)	65 (28.9%)	984 (25.9%)	
Migrant	2978 (74.0%)	160 (71.1%)	2818 (74.1%)	
Work Status:				<0.001
Employed	2040 (50.9%)	67 (30.2%)	1973 (52.1%)	
Unemployed	1970 (49.1%)	155 (69.8%)	1815 (47.9%)	
Lifestyle:				
Drinking^*^	459 (13.8%)	32 (18.2%)	427 (13.6%)	0.107
Smoking^*^	897 (27.0%)	65 (36.9%)	832 (26.5%)	0.003
Treatment Category:				<0.001
Initial	3625 (90.0%)	176 (78.2%)	3449 (90.7%)	
Retreat	402 (10.0%)	49 (21.8%)	353 (9.3%)	
TB-related symptoms:				
Cough^*^	2961 (73.5%)	167 (74.2%)	2794 (73.5%)	0.869
Expectoration^*^	2272 (56.4%)	144 (64.0%)	2128 (56.0%)	0.022
Hemoptysis^*^	721 (17.9%)	38 (16.9%)	683 (18.0%)	0.749
Chest Pain^*^	692 (17.2%)	30 (13.3%)	662 (17.4%)	0.138
Fever^*^	1414 (35.1%)	75 (33.3%)	1339 (35.2%)	0.614
Night Sweat^*^	332 (8.2%)	14 (6.2%)	318 (8.4%)	0.312
Fatigue^*^	625 (15.5%)	66 (29.3%)	559 (14.7%)	<0.001
Weight Loss^*^	1051 (26.1%)	83 (36.9%)	968 (25.5%)	<0.001
Comorbidities:				
Diabetes^*^	752 (18.7%)	69 (30.7%)	683 (18.0%)	<0.001
HBV^*^	429 (10.8%)	20 (9.01%)	409 (10.9%)	0.440
Hypertension^*^	419 (10.5%)	53 (23.9%)	366 (9.8%)	<0.001
Tumor^*^	152 (3.8%)	29 (13.1%)	123 (3.3%)	<0.001
Anemia^*^	382 (9.6%)	58 (26.2%)	324 (8.6%)	<0.001
EPTB^*^	2039 (50.6%)	104 (46.2%)	1935 (50.9%)	0.196
MTB0:				0.330
Positive	3917 (97.4%)	221 (98.7%)	3696 (97.4%)	
Negative	103 (2.6%)	3 (1.3%)	100 (2.6%)	
MTB2:				<0.001
Positive	280 (8.7%)	25 (20.8%)	255 (8.2%)	
Negative	2937 (91.3%)	95 (79.2%)	2842 (91.8%)	
Cavity:				0.003
No	2839 (78.8%)	138 (70.1%)	2701 (79.3%)	
Yes	766 (21.2%)	59 (29.9%)	707 (20.7%)	
DST:				
DS-TB^*^	2445 (86.2%)	123 (74.5%)	2322 (87.0%)	<0.001
DR-TB^*^	390 (13.8%)	42 (25.5%)	348 (13.0%)	<0.001
MDR-TB^*^	162 (5.7%)	24 (14.5%)	138 (5.2%)	<0.001
Hr-TB^*^	74 (2.6%)	7 (4.2%)	67 (2.5%)	0.200
RR-TB^*^	134 (4.7%)	11 (6.7%)	123 (4.6%)	0.307
Unknown^*^	1192 (29.6%)	60 (26.7%)	1132 (30.0%)	0.359

*A characteristic with two categories, where the ‘Yes’ category is shown in the table and the ‘No’ category is omitted. BMI, Body mass index; LOT, Length of treatment; HBV, Hepatitis B virus; EPTB, Extrapulmonary tuberculosis; MTB0, Bacteriological test result at the initiation of treatment; MTB2, Bacteriological test result at 2-month treatment; DST, Drug susceptibility testing; DS-TB, Drug-susceptible TB; DR-TB, Drug-resistant TB; MDR-TB, Multidrug-resistant TB; Hr-TB, Rifampicin-susceptible while isoniazid-resistant TB; RR-TB, Rifampicin-resistant TB.

**Figure 2:**
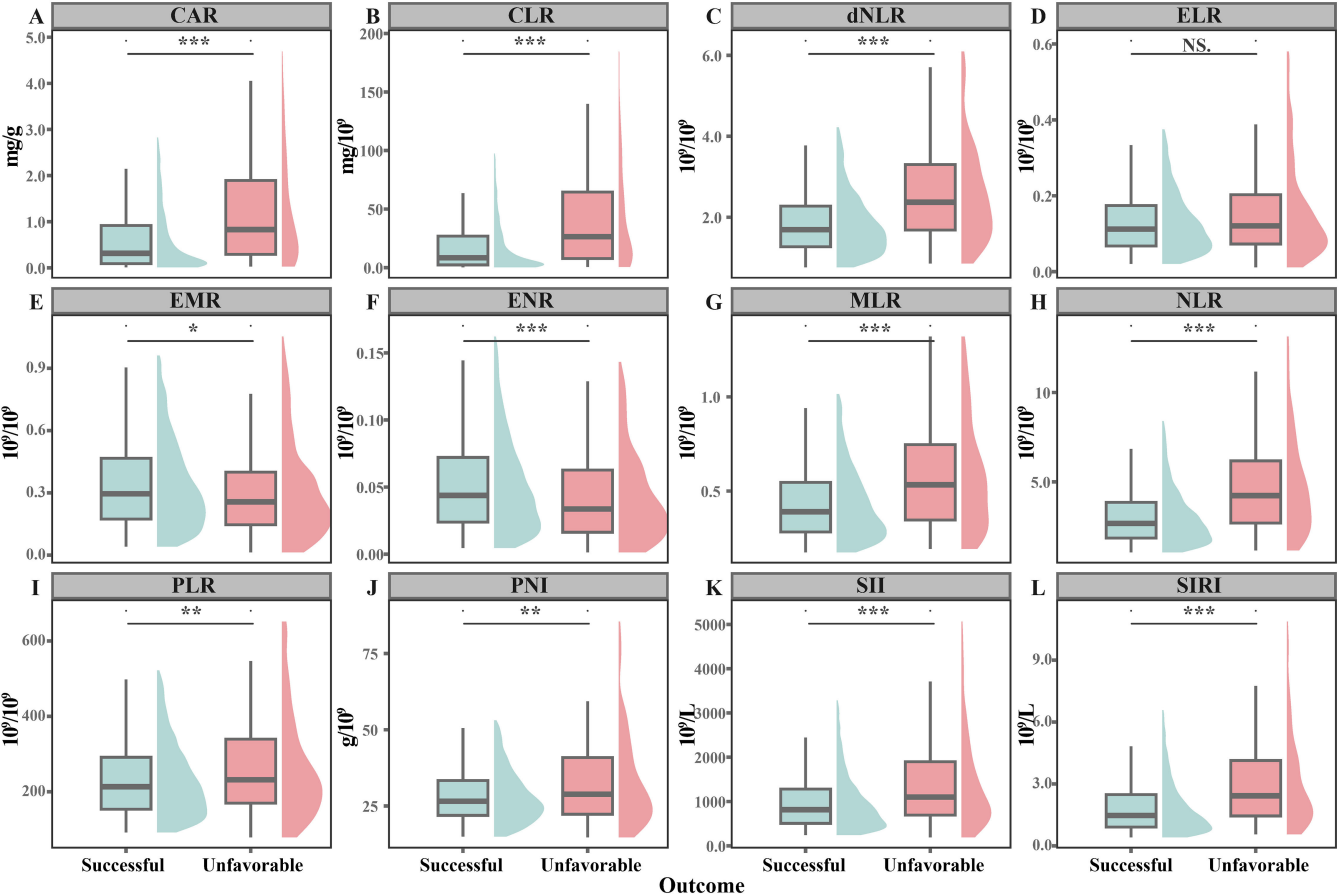
Difference in the distribution of inflammatory indices across patients with successful treatment and unfavorable outcome. *P<0.050; **P<0.010; ***P<0.001; ns, no significance. **(A)** CAR, C-reactive protein to albumin ratio; **(B)** CLR, C-reactive protein to lymphocyte ratio; **(C)** dNLR, Derived neutrophil to lymphocyte ratio; **(D)** ELR, Eosinophil to lymphocyte ratio; **(E)** EMR, Eosinophil to monocyte ratio; **(F)** ENR, Eosinophil to neutrophil ratio; **(G)** MLR, Monocyte to lymphocyte ratio; **(H)** NLR, Neutrophil to lymphocyte ratio; **(I)** PLR, Platelet to lymphocyte ratio; **(J)** PNI, Prognostic Nutritional Index; **(K)** SII, Systemic Immune-Inflammation Index; **(L)** SIRI, Systemic Inflammatory Response Index.

During 48991.79 person-months of follow-up, 225 cases with unfavorable outcome were identified (**Table 1**). These patients were more likely to be older (median: 58 vs 37 years, *P*<0.001), male (71.6% vs 61.6%, *P*=0.004), low body weight or overweight (56.9% vs 42.1%, *P*=0.001), unemployed (69.8% vs 47.9%, *P*<0.001), and retreatment cases (21.8% vs 9.3%, *P*<0.001) compared to those with successful treatment. They also exhibited higher rates of expectoration (64.0% vs 56.0%, *P*=0.022), fatigue (29.3% vs 14.7%, *P*<0.001), weight loss (36.9% vs 25.5%, *P*<0.001), and cavity (29.9% vs 20.7%, *P*=0.003). Additionally, higher prevalence of diabetes (30.7% vs 18.0%, *P*<0.001), hypertension (23.9% vs 9.8%, *P*<0.001), tumor (13.1% vs 3.3%, *P*<0.001), and anemia (26.2% vs 8.6%, *P*<0.001) were observed in this group. Bacteriological positivity at the 2-month treatment (20.8% vs 8.2%, *P*<0.001) and drug resistance rate (25.5% vs 13.0%, *P*<0.001) were significantly higher as well. Patients with unfavorable outcome had significantly higher median levels of CAR (0.83 vs 0.32, *P*<0.001), CLR (26.40 vs 8.45, *P*<0.001), dNLR (2.37 vs 1.69, *P*<0.001), MLR (0.53 vs 0.39, *P*<0.001), NLR (4.23 vs 2.67, *P*<0.001), PLR (232 vs 213, *P*=0.026), PNI (28.9 vs 26.5, *P*=0.004), SII (1110 vs 817, *P*<0.001), and SIRI (2.43 vs 1.48, *P*<0.001) compared to those with successful treatment, while the levels of EMR (0.26 vs 0.30, *P*=0.039) and ENR (0.03 vs 0.04, *P*=0.001) were notably lower (**Figure 2**).

### 3.2 Association between blood inflammatory status and unfavorable treatment outcome

**Figure 3:**
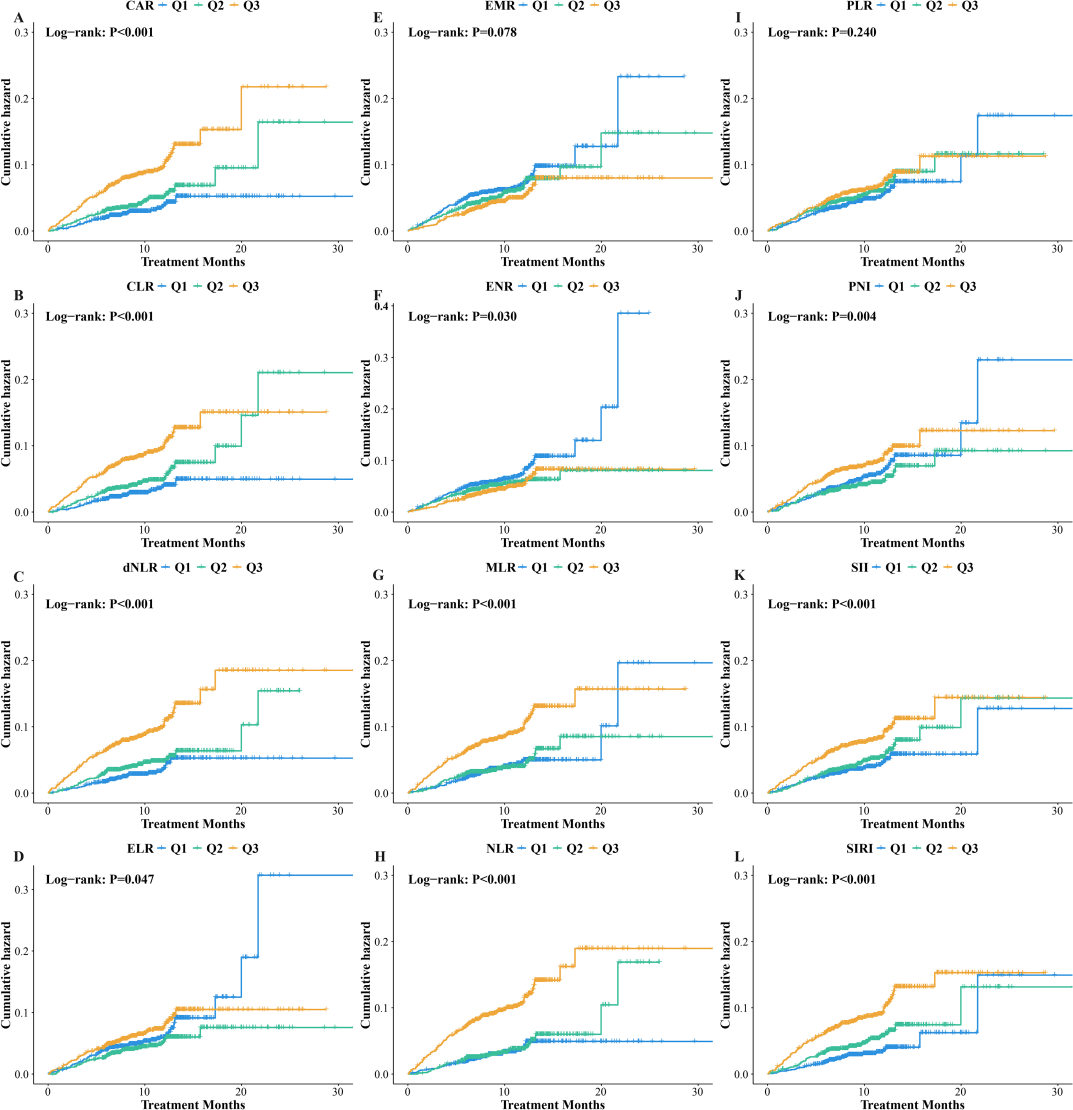
Kaplan-Meier analysis in different inflammatory status stratified by **(A)** CAR, C - reactive protein to albumin ratio; **(B)** CLR, C - reactive protein to lymphocyte ratio; **(C)** dNLR, Derived neutrophil to lymphocyte ratio; **(D)** ELR, Eosinophil to lymphocyte ratio; **(E)** EMR, Eosinophil to monocyte ratio; **(F)** ENR, Eosinophil to neutrophil ratio; **(G)** MLR, Monocyte to lymphocyte ratio; **(H)** NLR, Neutrophil to lymphocyte ratio; **(I)** PLR, Platelet to lymphocyte ratio; **(J)** PNI, Prognostic Nutritional Index; **(K)** SII, Systemic Immune - Inflammation Index; **(L)** SIRI, Systemic Inflammatory Response Index.

We observed significant differences in the cumulative incidence of unfavorable outcome across different inflammatory status, categorized by CAR (Log-rank *P*<0.001), CLR (Log-rank *P*<0.001), dNLR (Log-rank *P*<0.001), ELR (Log-rank *P*=0.047), ENR (Log-rank *P*=0.030), MLR (Log-rank *P*<0.001), NLR (Log-rank *P*<0.001), PNI (Log-rank *P*=0.004), SII (Log-rank *P*<0.001), and SIRI (Log-rank *P*<0.001) (**Figure 3**). Both unadjusted and adjusted Cox regression models (**Table 2**) indicated that the risk of unfavorable outcome progressively increased from Q1 to Q3 for CAR (*P* for trend=0.001), CLR (*P* for trend=0.004), dNLR (*P* for trend<0.001), NLR (*P* for trend<0.001), SII (*P* for trend=0.025), and SIRI (*P* for trend=0.007), while decreasing for ENR (*P* for trend=0.038). After adjusting for demographic and clinical characteristics, patients in the Q3 groups of CAR (HR:1.78, *P*=0.004), CLR (HR:1.72, *P*=0.007), dNLR (HR:1.99, *P*<0.001), NLR (HR:1.82, *P*=0.002), SII (HR:1.45, *P*=0.038), and SIRI (HR:1.62, *P*=0.014) had a 45% to 99% higher risk of unfavorable outcome compared to the Q1 groups. Conversely, the Q3 group of ENR (HR: 0.71, *P*=0.040) was associated with a 29% lower risk.

**Table 2:** Cox regression analysis between inflammatory status and unfavorable treatment outcome.

Inflammatory indices	Model 1		Model 2		Model 3	
	HR (95%*CI*)	P	HR (95%*CI*)	P	HR (95%*CI*)	P
CAR						
Continuous*	1.35 (1.26-1.45)	<0.001	1.24 (1.15-1.35)	<0.001	1.25 (1.15-1.36)	<0.001
Q1	ref		ref		ref	
Q2	1.44 (0.97-2.14)	0.069	1.16 (0.78-1.73)	0.472	1.15 (0.77-1.73)	0.501
Q3	2.84 (2.00-4.05)	<0.001	1.88 (1.30-2.71)	<0.001	1.78 (1.20-2.62)	0.004
*P* for trend		<0.001		<0.001		0.001
CLR						
Continuous*	1.16 (1.12-1.20)	<0.001	1.09 (1.04-1.14)	<0.001	1.11 (1.06-1.16)	<0.001
Q1	ref		ref		ref	
Q2	1.54 (1.04-2.29)	0.031	1.22 (0.82-1.81)	0.332	1.21 (0.81-1.82)	0.356
Q3	2.87 (2.01-4.10)	<0.001	1.82 (1.25-2.65)	0.002	1.72 (1.16-2.54)	0.007
*P* for trend		<0.001		<0.001		0.004
dNLR						
Continuous*	1.31 (1.24-1.39)	<0.001	1.19 (1.11-1.26)	<0.001	1.20 (1.12-1.29)	<0.001
Q1	ref		ref		ref	
Q2	1.44 (0.96-2.15)	0.075	1.17 (0.78-1.75)	0.449	1.17 (0.78-1.76)	0.440
Q3	2.98 (2.09-4.25)	<0.001	2.10 (1.46-3.02)	<0.001	1.99 (1.38-2.89)	<0.001
*P* for trend		<0.001		<0.001		<0.001
ELR						
Continuous*	1.13 (1.05-1.21)	<0.001	1.01 (0.92-1.12)	0.774	0.99 (0.89-1.09)	0.800
Q1	ref		ref		ref	
Q2	0.79 (0.56-1.11)	0.170	0.77 (0.55-1.09)	0.139	0.85 (0.60-1.19)	0.342
Q3	1.19 (0.87-1.61)	0.276	0.87 (0.64-1.19)	0.379	0.88 (0.64-1.21)	0.434
*P* for trend		0.243		0.420		0.453
EMR						
Continuous*	0.84 (0.59-1.19)	0.323	0.80 (0.57-1.11)	0.185	0.80 (0.58-1.10)	0.169
Q1	ref		ref		ref	
Q2	0.87 (0.64-1.18)	0.374	0.90 (0.66-1.23)	0.516	0.97 (0.71-1.32)	0.842
Q3	0.69 (0.50-0.95)	0.025	0.71 (0.51-0.99)	0.041	0.74 (0.53-1.03)	0.071
*P* for trend		0.025		0.043		0.077
ENR						
Continuous*	0.82 (0.69-0.98)	0.032	0.81 (0.69-0.96)	0.015	0.82 (0.70-0.97)	0.020
Q1	ref		ref		ref	
Q2	0.75 (0.55-1.02)	0.068	0.75 (0.55-1.03)	0.079	0.81 (0.59-1.11)	0.183
Q3	0.67 (0.48-0.92)	0.013	0.67 (0.49-0.93)	0.016	0.71 (0.51-0.99)	0.040
*P* for trend		0.011		0.014		0.038
MLR						
Continuous*	1.36 (1.26-1.47)	<0.001	1.19 (1.09-1.30)	<0.001	1.16 (1.05-1.28)	0.003
Q1	ref		ref		ref	
Q2	1.01 (0.69-1.49)	0.961	0.80 (0.54-1.19)	0.273	0.77 (0.52-1.14)	0.196
Q3	2.28 (1.64-3.16)	<0.001	1.38 (0.97-1.96)	0.070	1.21 (0.84-1.74)	0.300
*P* for trend		<0.001		0.021		0.141
NLR						
Continuous*	1.16 (1.12-1.21)	<0.001	1.08 (1.04-1.13)	<0.001	1.09 (1.04-1.15)	<0.001
Q1	ref		ref		ref	
Q2	1.10 (0.73-1.66)	0.656	0.88 (0.58-1.33)	0.535	0.91 (0.60-1.38)	0.643
Q3	3.06 (2.17-4.33)	<0.001	1.96 (1.36-2.81)	<0.001	1.82 (1.25-2.63)	0.002
*P* for trend		<0.001		<0.001		<0.001
PLR						
Continuous*	1.15 (1.06-1.24)	<0.001	1.04 (0.95-1.14)	0.369	1.03 (0.93-1.14)	0.580
Q1	ref		ref		ref	
Q2	1.16 (0.83-1.63)	0.384	1.09 (0.77-1.53)	0.632	1.10 (0.78-1.56)	0.571
Q3	1.32 (0.96-1.83)	0.091	1.01 (0.72-1.42)	0.954	0.94 (0.66-1.34)	0.751
*P* for trend		0.090		0.990		0.703
PNI						
Continuous*	1.18 (1.12-1.25)	<0.001	1.08 (1.01-1.15)	0.022	1.09 (1.01-1.17)	0.023
Q1	ref		ref		ref	
Q2	0.81 (0.57-1.15)	0.230	0.77 (0.54-1.09)	0.139	0.80 (0.56-1.14)	0.210
Q3	1.37 (1.01-1.86)	0.046	1.01 (0.73-1.38)	0.963	1.02 (0.74-1.41)	0.903
*P* for trend		0.031		0.847		0.813
SII						
Continuous*	1.21 (1.13-1.30)	<0.001	1.12 (1.04-1.19)	0.001	1.12 (1.04-1.21)	0.003
Q1	ref		ref		ref	
Q2	1.22 (0.85-1.76)	0.284	1.01 (0.70-1.46)	0.975	1.06 (0.73-1.54)	0.749
Q3	1.99 (1.43-2.78)	<0.001	1.56 (1.11-2.19)	0.011	1.45 (1.02-2.07)	0.038
*P* for trend		<0.001		0.005		0.025
SIRI						
Continuous*	1.21 (1.15-1.27)	<0.001	1.14 (1.08-1.20)	<0.001	1.15 (1.08-1.22)	<0.001
Q1	ref		ref		ref	
Q2	1.58 (1.07-2.35)	0.022	1.21 (0.81-1.81)	0.341	1.16 (0.78-1.74)	0.462
Q3	2.81 (1.96-4.02)	<0.001	1.86 (1.28-2.70)	0.001	1.62 (1.10-2.37)	0.014
*P* for trend		<0.001		<0.001		0.007

*for each one standard deviation (SD) increase. Model 1: Non-adjusted model; Model 2 adjusted for: Age, Sex, and BMI; Model 3 adjusted for: Age, Sex, BMI, Work Status, Smoking, Treatment Category, Expectoration, Fatigue, Weight Loss, Diabetes, Hypertension, Tumor, Anemia, EPTB, cavity, DST, and MTB2. BMI, Body Mass Index; EPTB, Extrapulmonary tuberculosis; DST, Drug susceptibility testing; MTB2, Bacteriological test result at 2-month treatment; CAR, C-reactive protein to albumin ratio; CLR, C-reactive protein to lymphocyte ratio; dNLR, Derived neutrophil to lymphocyte ratio; ELR, Eosinophil to lymphocyte ratio; EMR, Eosinophil to monocyte ratio; ENR, Eosinophil to neutrophil ratio; MLR, Monocyte to lymphocyte ratio; NLR, Neutrophil to lymphocyte ratio; PLR, Platelet to lymphocyte ratio; PNI, Prognostic Nutritional Index; SII, Systemic Immune - Inflammation Index; SIRI, Systemic Inflammatory Response Index.

**Figure 4:**
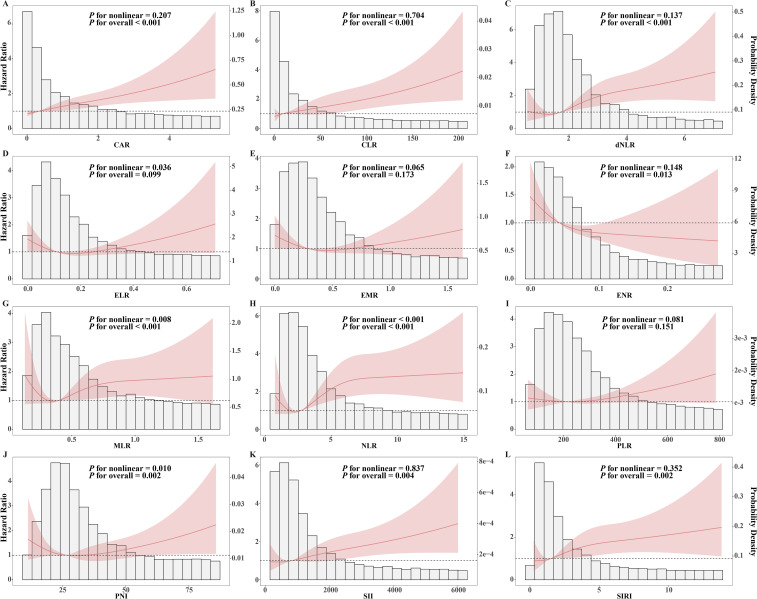
RCS analysis between inflammatory indices and unfavorable treatment outcome. The red line and shaded area represent the Hazard Ratio and its 95% confidence interval, while the bars represent the probability density. **(A)** CAR, C - reactive protein to albumin ratio; **(B)** CLR, C - reactive protein to lymphocyte ratio; **(C)** dNLR, Derived neutrophil to lymphocyte ratio; **(D)** ELR, Eosinophil to lymphocyte ratio; **(E)** EMR, Eosinophil to monocyte ratio; **(F)** ENR, Eosinophil to neutrophil ratio; **(G)** MLR, Monocyte to lymphocyte ratio; **(H)** NLR, Neutrophil to lymphocyte ratio; **(I)** PLR, Platelet to lymphocyte ratio; **(J)** PNI, Prognostic Nutritional Index; **(K)** SII, Systemic Immune - Inflammation Index; **(L)** SIRI, Systemic Inflammatory Response Index.

The RCS curves was employed to examine the potential non-linear relationship between inflammatory indices and unfavorable outcome. (**Figure 4**). A positive linear association was observed between unfavorable outcome and CAR (*P* for nonlinearity=0.207, *P* for overall<0.001), CLR (*P* for nonlinearity=0.704, *P* for overall<0.001), dNLR (*P* for nonlinearity=0.137, *P* for overall<0.001), SII (*P* for nonlinearity=0.837, *P* for overall=0.004), and SIRI (*P* for nonlinearity=0.352, *P* for overall=0.002), while a linear negative association was noted with ENR (*P* for nonlinearity=0.148, *P* for overall=0.013). After adjusting for covariates (**Table 2**), each 1-SD increase in the former indices were associated with a 11% to 25% higher risk of unfavorable outcome (HR: 1.11–1.25, all *P*<0.050), whereas each 1-SD increase in ENR reduced the risk by 18% (HR: 0.82, *P*=0.020). Additionally, non-linear relationships were found between unfavorable outcome risk and MLR (*P* for nonlinearity=0.008, *P* for overall<0.001), NLR (*P* for nonlinearity<0.001, *P* for overall<0.001), and PNI (*P* for nonlinearity=0.010, *P* for overall=0.002).

### 3.3 Subgroup analysis

Subgroup analyses were conducted to further explore the influence of demographic and clinical characteristics on the association between inflammatory status and unfavorable outcome (**Supplementary Tables 5**-**16**). The results indicated that the association differed by age, sex, BMI, DST and comorbidities. In the primary results and most subgroups, elevated levels of CAR, CLR, dNLR, NLR, SII, and SIRI significantly increased the risk of unfavorable outcome. However, this effect was absent in individuals younger than 30 years, female, susceptible cases, those without cavity and those with hepatitis B viral infections or diabetes. Furthermore, in the primary results, none of the indicators in the Q2 group significantly impacted treatment outcome. However, in patients aged 30–60 years, male, those with a BMI≥24.0 kg/m², smokers, retreatment cases, and individuals with tumor, the Q2 groups of CAR, CLR, dNLR, and SII were associated with a significantly increased risk of unfavorable outcome. Additionally, EMR and ENR were identified as protective factors for treatment outcomes, with their effects significantly moderated by BMI (all *P* for interaction<0.050).

### 3.4 Predictive value of blood inflammatory status for treatment outcome

**Figure 5:**
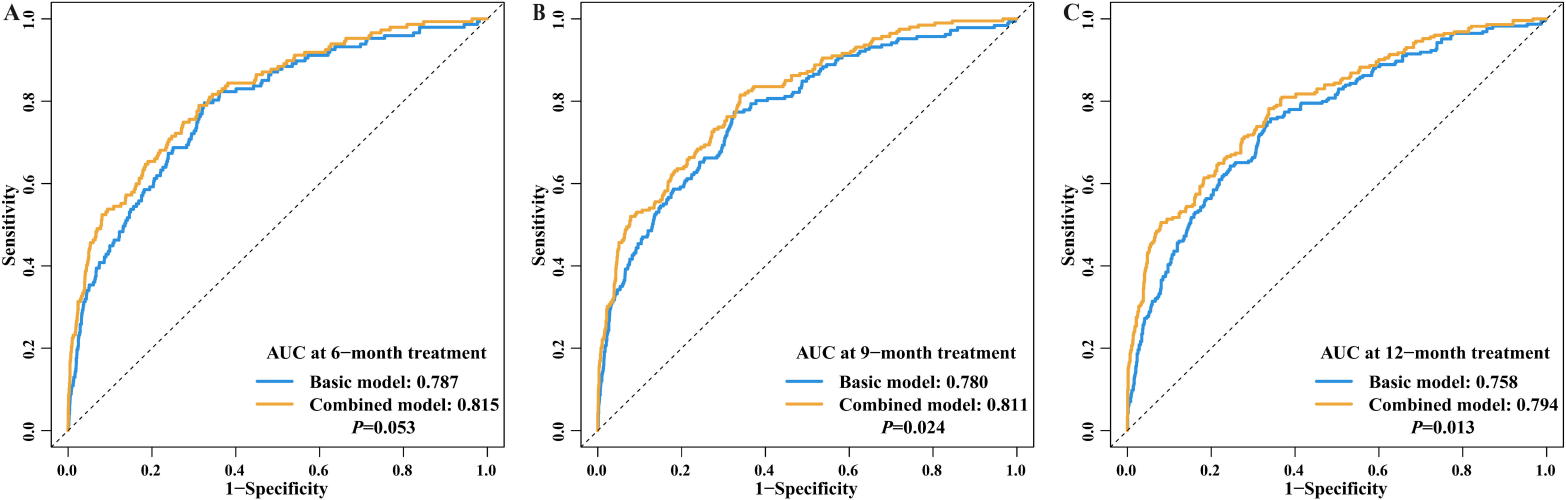
Predictive value for treatment outcome between basic model and combined model. **(A)** AUC at 6-month treatment; **(B)** AUC at 9-month treatment; **(C)** AUC at 12-month treatment Note: Basic model: included age, sex, BMI, work status, smoking, treatment category, expectoration, fatigue, weight loss, diabetes, hypertension, tumor, anemia, EPTB, cavity, DST, and MTB2. Combined model: a combination of blood inflammatory indices, added to the characteristics included in the basic model. BMI, Body Mass Index; EPTB, Extrapulmonary tuberculosis; DST, Drug susceptibility testing; MTB2, Bacteriological test result at 2-month treatment; AUC, Area under curve.

The ROC analysis demonstrated the predictive value of inflammatory indices for the prognosis of pulmonary tuberculosis patients. The area under the curve (AUC) for each blood inflammatory index in predicting treatment outcome at 6-, 9-, and 12-month treatment was 0.785-0.804, 0.781–0.793, and 0.762–0.773, respectively (**Table 3**, **Supplementary Figure 1**), indicating good and stable predictive performances over a general treatment course for pulmonary tuberculosis. Importantly, compared to the baseline model without blood inflammatory status, the combination of the inflammatory status significantly improved the model’s predictive accuracy (9-month AUC: 0.811 vs 0.780, *P*=0.024; 12-month AUC: 0.794 vs 0.758, *P*=0.013, **Table 3**, **Figure 5**).

**Table 3:** Area under the curve for ROC analysis.

Model	6-month AUC	*P*	9-month AUC	*P*	12-month AUC	*P*
Basic model	0.787	ref	0.780	ref	0.758	ref
+CAR	0.796	0.460	0.793	0.287	0.770	0.319
+CLR	0.797	0.409	0.793	0.259	0.770	0.318
+dNLR	0.804	0.069	0.791	0.407	0.770	0.313
+ELR	0.789	0.881	0.782	0.865	0.765	0.540
+EMR	0.788	0.922	0.781	0.927	0.763	0.596
+ENR	0.792	0.635	0.782	0.853	0.762	0.697
+MLR	0.785	0.919	0.788	0.479	0.773	0.137
+NLR	0.803	0.107	0.793	0.246	0.771	0.217
+PLR	0.789	0.869	0.783	0.713	0.767	0.450
+PNI	0.794	0.552	0.791	0.396	0.772	0.202
+SII	0.795	0.482	0.788	0.471	0.765	0.502
+SIRI	0.790	0.782	0.782	0.797	0.763	0.559
Combined model	0.815	0.053	0.811	0.024	0.794	0.013

Basic model: included age, sex, BMI, work status, smoking, treatment category, expectoration, fatigue, weight loss, diabetes, hypertension, tumor, anemia, EPTB, cavity, DST, and MTB2. Combined model: a combination of serum inflammatory indices, added to the characteristics included in the basic model. BMI, Body Mass Index; EPTB, Extrapulmonary tuberculosis; DST, Drug susceptibility testing; MTB2, Bacteriological test result at 2-month treatment; AUC, Area under curve. CAR, C-reactive protein to albumin ratio; CLR, C-reactive protein to lymphocyte ratio; dNLR, Derived neutrophil to lymphocyte ratio; ELR, Eosinophil to lymphocyte ratio; EMR, Eosinophil to monocyte ratio; ENR, Eosinophil to neutrophil ratio; MLR, Monocyte to lymphocyte ratio; NLR, Neutrophil to lymphocyte ratio; PLR, Platelet to lymphocyte ratio; PNI, Prognostic Nutritional Index; SII, Systemic Immune - Inflammation Index; SIRI, Systemic Inflammatory Response Index.

### 3.5 Sensitivity analysis

Sensitivity analyses were conducted to evaluate the robustness of the results. Whether excluding patients with comorbidities or performing a complete case analysis, Kaplan-Meier analysis (**Supplementary Figures 2**-**3**) and Cox regression analysis (**Supplementary Tables 17**-**18**) indicated that CAR, CLR, dNLR, NLR, and SIRI remained significant risk factors for unfavorable outcome (all *P*<0.05). The patterns of association between each inflammatory index and the risk of unfavorable outcome demonstrated by RCS in both sensitivity analyses (**Supplementary Figures 4**-**5**) were also consistent with the primary results. The AUC of the combined model further improved to 0.852 (*P*=0.001), 0.830 (*P*=0.001), and 0.782 (*P*=0.032) at 6, 9, 12-month treatment respectively in the population without comorbidities (**Supplementary Figure 6**, **Supplementary Table 19**) and to 0.803 (*P*=0.024), 0.797 (*P*=0.035), and 0.766 (*P*=0.017) in complete case analysis (**Supplementary Figure 7**, **Supplementary Table 20**), both outperforming the basic model as indicated by the primary results.

## 4 Discussion

TB is a chronic infectious disease caused by *Mycobacterium tuberculosis* (Mtb), whose onset and progression are intricately linked to immune cell-mediated inflammatory responses. This study systematically investigated the association between various blood inflammatory indices and the risk of unfavorable prognosis in pulmonary tuberculosis and identified three major findings. First, this study revealed that blood inflammatory status is an independent prognostic factor in TB patients, with both linear and non-linear associations observed. Second, subgroup analyses indicated that the association between blood inflammatory status and unfavorable outcome varied by age, sex, BMI, and comorbidities. Third, ROC analysis demonstrated that blood inflammatory status reliably predicts unfavorable outcome and significantly improves the model’s predictive accuracy. These findings provide valuable insights for advancing personalized treatment strategies in TB management.

This study identified that elevated levels of CAR, CLR, dNLR, NLR, SII, and SIRI significantly increased the risk of unfavorable prognosis, whereas ENR emerged as a protective factor, providing additional support and validation for existing evidence. A multicenter study in Kenya reported that elevated CRP levels, combined with malnutrition, increased the short-term mortality rate in HIV-positive TB patients by 5.6-fold (12). CRP is the most widely used blood marker for assessing systemic inflammation, while albumin, a visceral protein with anti-inflammatory and antioxidant properties, is the most valuable indicator for assessing nutritional status (31). Thus, CAR represents the comprehensive impact of inflammatory status and nutritional condition on prognosis. Similarly, a clinical cohort study in Wuhan, China, found that elevated levels of CAR, CLR, and NLR were significant risk factors for mortality in RR/MDR-TB patients (14). The calculations for CLR and NLR involve immune cells, specifically neutrophils and lymphocytes. Mtb can induce neutrophil necrosis, which not only promotes bacterial growth within phagocytes but also accelerates caseous necrosis and tissue liquefaction (32, 33). Therefore, neutrophil infiltration is a significant feature of tuberculosis progression (32–36). Conversely, lymphopenia is commonly observed in severely ill patients (37–39), and the lymphocyte proportion below 16% increased the risk of treatment failure by 11-folds. Additionally, this study is the first to explore the impact of ENR, SII, and SIRI on the risk of unfavorable prognosis in pulmonary tuberculosis. Consistent with other biomarkers, elevated levels of SII and SIRI also significantly increased the unfavorable outcome risk. Interestingly, elevated ENR levels were associated with a protective effect, possibly supporting recent findings that eosinophils play a critical role in the early control of Mtb infection in animal models (40, 41).

Our study revealed that CAR, CLR, dNLR, ENR, SII, and SIRI are linearly associated with the risk of unfavorable outcome in TB patients, while MLR, NLR, and PNI exhibit U-shaped relationships surprisingly. These nonlinear associations may reflect the dual roles of neutrophil- and monocyte-mediated inflammation and nutritional status. Elevated NLR and MLR indicate a hyperactive immune response with increased neutrophils and monocytes and/or decreased lymphocytes, which have been linked to poor TB treatment outcomes (13, 14, 42, 43). In chronic or poorly controlled TB, extensive infiltration of neutrophils releases substances such as MMPs, S100A8/A9, and proteases, contributing to tissue damage and lung dysfunction (44). Meanwhile, monocytes, influenced by Mtb, may differentiate into the M2 macrophage phenotype, impairing antigen presentation and T-cell activation (45). Low MLR and NLR, conversely, may reflect impaired innate immune function. Neutrophils and monocytes, as key cells in innate immunity, suppress Mtb growth through phagocytosis, oxidative burst, and induction of adaptive immunity during early stage of infection (46, 47). PNI is a critical index of nutritional and immune status. Low PNI indicates malnutrition, characterized by low albumin, and/or impairment of adaptive immunity, marked by lymphopenia, and is associated with more severe manifestations and poorer anti-TB treatment outcome (48, 49). The association between high PNI and unfavorable outcomes may be influenced by comorbidities. In our sensitivity analysis, excluding patients with comorbidities rendered this association nonsignificant. Overall, characterizing these nonlinear associations through RCS analysis is essential for identifying inflection points where inflammation transitions from being protective to harmful. These insights support threshold-based risk stratification and the development of personalized TB therapeutic strategies tailored to individual inflammatory profiles.

This study further identified that traditionally recognized high-risk groups—older adults, males, individuals with low BMI, and those with comorbidities (50–52)—experience an increased inflammation-mediated unfavorable prognosis risk and/or the harmful effects of inflammatory factors occurring at lower doses, suggesting that these populations may be key beneficiaries of host-directed therapy (HDT). HDT has emerged as a promising approach in tuberculosis treatment, aiming to shorten treatment duration, improve outcomes of drug-resistant cases, and mitigate immunopathology (53). However, its application is limited by the narrow therapeutic windows of many critical immune targets and the considerable clinical phenotypic heterogeneity among individuals (54). The significant interactions between age, sex, BMI, and blood inflammatory status observed in this study underscore this heterogeneity. Therefore, precise disease phenotype analysis and identification of tuberculosis subtypes are essential for developing subtype-specific HDT strategies (55).

In this study, the AUC for predicting treatment outcome at 6-, 9-, and 12- month post-treatment based on blood inflammatory status ranged from 0.785–0.804, 0.781–0.793, and 0.762–0.773, respectively, demonstrating stable and comparable predictive performance to previous studies (14, 56). Although the baseline model consists of well-established prognostic characteristics in tuberculosis, including age, sex, residency, smoking status, chronic comorbidities, and drug resistance types (50–52), the addition of inflammatory indices significantly improved the model’s predictive accuracy, indicating that demographic and clinical characteristics alone may not fully address the needs for personalized TB treatment, as observed in other diseases (14, 56, 57). Therefore, clinical practice should incorporate pretreatment assessment of blood inflammatory status to enhance the accuracy of prognosis prediction and guide personalized tuberculosis treatment strategies.

Several limitations may affect the interpretation of our results. First, despite a relatively large sample, the generalizability of the findings is limited, necessitating further studies to enhance the external validity of the results. Second, although numerous covariates were adjusted for, there may still be unknown risk factors not captured by the hospital information system. Third, this study focused on the baseline inflammatory status without examining longitudinal changes, which future research could assess the impact of inflammatory trajectories on treatment outcomes through a prospective study design.

## 5 Conclusion

Our study delineated the potential non-linear relationship between blood inflammatory indices and unfavorable outcome in PTB and identified elevated CAR, CLR, dNLR, NLR, SII, and SIRI as independent risk factors. Furthermore, we demonstrated that the combination of inflammatory indices significantly improved the prognostic value of the basic model incorporating only demographic and clinical features. Therefore, clinical practice should leverage the value of pretreatment inflammatory status assessments, and future research should explore their applications in personalized therapy.

## Data Availability

The raw data supporting the conclusions of this article will be made available by the authors, without undue reservation.
