# Supplementary material for: Association between blood inflammatory status and the survival of tuberculosis: a five-year cohort study
Source: Front Immunol. 2025 Mar 21;16:1556857. doi: 10.3389/fimmu.2025.1556857 (PMC11968758; doi:10.3389/fimmu.2025.1556857)
Supplement: Supplementary file 1 [file Table1.docx]

## Supplementary material Association between blood inflammatory status and the survival of tuberculosis: a five-year cohort study

Supplementary Table 1 Definitions of TB treatment outcomes

| Treatment  Outcomes | Definitions |
| --- | --- |
| Successful treatment | (i) Cured: A pulmonary TB patient with bacteriologically confirmed TB at the beginning of treatment who completed treatment as recommended by the national policy with evidence of bacteriological response and no evidence of failure. (ii)Treatment completed: A patient who completed treatment as recommended by the national policy whose outcome does not meet the definition of cure or treatment failure. |
| Unfavorable  outcome | (i) Treatment failure: A patient whose treatment regimen needed to be terminated or permanently changed^¶^ to a new regimen or treatment strategy. (ii) Died: A patient who died before starting treatment or during treatment. |

Note: ¶: Reasons for the change include: no clinical response and/or no bacteriological response; adverse drug reactions; or evidence of additional drug resistance to medicines in the regimen(1).

Supplementary Table 2 Missing data overview of the covariates

| Covariables | Number of missing | Percentage of missing (%) |
| --- | --- | --- |
| MTB0 | 7 | 0.17 |
| HBV | 54 | 1.34 |
| Hypertension | 55 | 1.37 |
| Tumor | 55 | 1.37 |
| Anemia | 56 | 1.39 |
| Patient Delay | 81 | 2.01 |
| Hospital Delay | 81 | 2.01 |
| Total Delay | 81 | 2.01 |
| Drinking | 705 | 17.51 |
| Smoking | 706 | 17.53 |
| BMI | 747 | 18.55 |
| MTB2 | 810 | 20.11 |
| Cavity | 422 | 10.48 |
| DST | 1192 | 29.60 |

Note: MTB0: Bacteriological test result at the initiation of treatment; HBV: Hepatitis B virus; EPTB: Extrapulmonary tuberculosis; BMI: Body mass index; MTB2: Bacteriological test result at 2-month treatment; DST: Drug susceptibility testing

Supplementary Table 3 The 33rd and 66th percentile of each inflammation index

| Inflammation index | P33 | P66 | |  |
| --- | --- | --- | --- | --- |
| CAR/mg•g^-1^ | 0.14 | 0.72 | |  |
| CLR/mg•10^-9^ | 3.51 | 20.81 | |  |
| dNLR/10^9^•10^-9^ | 1.39 | 2.1 | |  |
| ELR/10^9^•10^-9^ | 0.08 | 0.15 | |  |
| EMR/10^9^•10^-9^ | 0.2 | 0.4 | |  |
| ENR/10^9^•10^-9^ | 0.03 | 0.06 | | |
| MLR/10^9^•10^-9^ | 0.31 | 0.5 | | |
| NLR/10^9^•10^-9^ | 2.08 | 3.49 | | |
| PLR/10^9^•10^-9^ | 169.8 | 264.81 | | |
| PNI/g•10^-9^ | 23.05 | 31.2 | | |
| SII/10^9^•L^-1^ | 586.51 | 1122.03 |  |  |
| SIRI/10^9^•L^-1^ | 1.05 | 2.17 |  |  |

Note: P33: The 33rd percentile, P66: The 66th percentile. CAR: C-reactive protein to albumin ratio; CLR: C-reactive protein to lymphocyte ratio; dNLR: Derived neutrophil to lymphocyte ratio; ELR: Eosinophil to lymphocyte ratio; EMR: Eosinophil to monocyte ratio; ENR: Eosinophil to neutrophil ratio; MLR: Monocyte to lymphocyte ratio; NLR: Neutrophil to lymphocyte ratio; PLR: Platelet to lymphocyte ratio; PNI: Prognostic Nutritional Index; SII: Systemic Immune-Inflammation Index; SIRI: Systemic Inflammatory Response Index.

.

Supplementary Table 4 Univariate cox regression analysis of demographic and clinical characteristics associated with unfavorable treatment outcomes in three analyses

| Characteristics | Main Analysis | | Sensitivity Analysis 1 | | Sensitivity Analysis 2 | |
| --- | --- | --- | --- | --- | --- | --- |
|  | HR (95% *CI*) | *P* | HR (95% *CI*) | *P* | HR (95% *CI*) | *P* |
| Age/years | 1.04 (1.04-1.05) | <0.001 | 1.03 (1.02-1.05) | <0.001 | 1.04 (1.04-1.05) | <0.001 |
| Total Delay/month | 1.02 (0.98-1.07) | 0.328 | 0.95 (0.85-1.06) | 0.126 | 1.02 (0.97-1.07) | 0.353 |
| Patient Delay/month | 1.01 (0.96-1.07) | 0.592 | 0.94 (0.83-1.08) | 0.372 | 1.01 (0.96-1.07) | 0.640 |
| Hospital Delay/month | 1.04 (0.91-1.18) | 0.568 | 0.98 (0.78-1.25) | 0.394 | 1.04 (0.90-1.20) | 0.606 |
| Sex: |  |  |  |  |  |  |
| Female | ref |  | ref |  | ref |  |
| Male | 1.56 (1.17-2.09) | 0.003 | 1.49 (0.96-2.33) | 0.078 | 1.58 (1.12-2.22) | 0.009 |
| BMI: /kg·m^-2^ |  |  |  |  |  |  |
| <18.5 | ref |  | ref |  | ref |  |
| 18.5~24.0 | 0.73 (0.55-0.98) | 0.033 | 0.91 (0.57-1.44) | 0.687 | 0.77 (0.55-1.08) | 0.126 |
| >24.0 | 1.22 (0.82-1.82) | 0.325 | 1.31 (0.60-2.86) | 0.498 | 1.23 (0.76-1.97) | 0.403 |
| Domicile: |  |  |  |  |  |  |
| Resident | ref |  | ref |  | ref |  |
| Migrant | 0.84 (0.63-1.13) | 0.252 | 0.93 (0.57-1.52) | 0.762 | 0.75 (0.54-1.05) | 0.097 |
| Work Status: |  |  |  |  |  |  |
| Employed | ref |  | ref |  | ref |  |
| Unemployed | 2.51 (1.88-3.34) | <0.001 | 2.15 (1.38-3.35) | <0.001 | 2.61 (1.85-3.67) | <0.001 |
| Drinking: |  |  |  |  |  |  |
| No | ref |  | ref |  | ref |  |
| Yes | 1.42 (0.99-2.03) | 0.054 | 2.34 (1.36-4.03) | 0.002 | 1.37 (0.90-2.09) | 0.139 |
| Smoking: |  |  |  |  |  |  |
| No | ref |  | ref |  | ref |  |
| Yes | 1.66 (1.26-2.19) | <0.001 | 2.35 (1.51-3.68) | <0.001 | 1.67 (1.21-2.31) | 0.002 |
| Treatment Category: |  |  |  |  |  |  |
| Initial | ref |  | ref |  | ref |  |
| Retreat | 2.31 (1.68-3.18) | <0.001 | 3.18 (1.92-5.26) | <0.001 | 2.26 (1.57-3.27) | <0.001 |
| TB-related symptoms: |  |  |  |  |  |  |
| Cough | 1.03 (0.76-1.39) | 0.854 | 1.07 (0.66-1.73) | 0.798 | 1.01 (0.70-1.47) | 0.948 |
| Expectoration | 1.36 (1.04-1.79) | 0.026 | 1.22 (0.79-1.88) | 0.366 | 1.38 (0.98-1.95) | 0.067 |
| Hemoptysis | 0.99 (0.70-1.41) | 0.970 | 1.08 (0.62-1.89) | 0.780 | 1.18 (0.81-1.73) | 0.385 |
| Chest Pain | 0.73 (0.50-1.08) | 0.112 | 1.01 (0.60-1.70) | 0.978 | 0.77 (0.46-1.30) | 0.328 |
| Fever | 0.91 (0.69-1.20) | 0.499 | 0.95 (0.61-1.49) | 0.824 | 1.03 (0.74-1.44) | 0.851 |
| Night Sweat | 0.73 (0.42-1.25) | 0.248 | 0.86 (0.38-1.98) | 0.730 | 0.85 (0.47-1.53) | 0.593 |
| Fatigue | 2.30 (1.73-3.06) | <0.001 | 1.47 (0.84-2.57) | 0.175 | 2.44 (1.75-3.40) | <0.001 |
| Weight Loss | 1.64 (1.25-2.15) | <0.001 | 1.13 (0.69-1.87) | 0.623 | 1.67 (1.22-2.29) | 0.002 |
| Comorbidities: |  |  |  |  |  |  |
| Diabetes | 1.97 (1.48-2.61) | <0.001 | - | - | 1.85 (1.33-2.58) | <0.001 |
| HBV | 0.79 (0.50-1.25) | 0.313 | - | - | 0.61 (0.34-1.09) | 0.094 |
| Hypertension | 2.79 (2.05-3.80) | <0.001 | - | - | 3.20 (2.25-4.55) | <0.001 |
| Tumor | 4.12 (2.79-6.08) | <0.001 | - | - | 3.32 (2.06-5.37) | <0.001 |
| Anemia | 3.44 (2.56-4.63) | <0.001 | - | - | 3.47 (2.46-4.90) | <0.001 |
| EPTB: |  |  |  |  |  |  |
| No | ref |  | ref |  | ref |  |
| Yes | 0.72 (0.55-0.94) | 0.015 | 0.69 (0.45-1.06) | 0.093 | 0.87 (0.64-1.19) | 0.377 |
| MTB0: |  |  |  |  |  |  |
| Negative | ref |  | ref |  | ref |  |
| Positive | 1.98 (0.64-6.20) | 0.238 | 0.74 (0.23-2.35) | 0.614 | 1.48 (0.30-8.83) | 0.993 |
| MTB2: |  |  |  |  |  |  |
| Negative | ref |  | ref |  | ref |  |
| Positive | 1.64 (1.12-2.42) | 0.012 | 1.87 (0.99-3.52) | 0.054 | 1.81 (1.20-2.73) | 0.004 |
| Cavity: |  |  |  |  |  |  |
| No | ref |  | ref |  | ref |  |
| Yes | 1.57 (1.18-2.09) | 0.002 | 1.69 (1.02-2.79) | 0.041 | 1.41 (1.01-1.97) | 0.043 |
| DST: |  |  |  |  |  |  |
| DS-TB | ref |  | ref |  | ref |  |
| MDR-TB | 2.15 (1.39-3.31) | <0.001 | 3.72 (2.03-6.80) | <0.001 | 2.34 (1.47-3.74) | <0.001 |
| DR_nonMDR | 1.24 (0.76-2.02) | 0.387 | 1.35 (0.62-2.96) | 0.446 | 1.51 (0.92-2.48) | 0.104 |

Note: Main Analysis: Conducted on the data after performing multiple imputation for missing values. Sensitivity Analysis 1: Conducted on the data after performing multiple imputation and excluding patients with comorbidities. Sensitivity Analysis 2: Conducted on the data after excluding patients with missing data. BMI: Body mass index; HBV: Hepatitis B virus; EPTB: Extrapulmonary tuberculosis; MTB0: Bacteriological test result at the initiation of treatment; MTB2: Bacteriological test result at 2-month treatment; DST: Drug susceptibility testing; DS-TB: Drug-susceptible; MDR-TB: Multidrug-resistant TB; DR_nonMDR: Drug-resistant TB except MDR-TB.

Supplementary Table 5 Subgroup analysis of the association between CAR and unfavorable treatment outcome

| Subgroup | HR (95%*CI*) | | | | | *P* for  interaction |
| --- | --- | --- | --- | --- | --- | --- |
|  | Q1 | Q2 | *P* | Q3 | *P* |  |
| Age group/years |  |  |  |  |  | 0.045 |
| <30 | ref | 0.64 (0.22-1.80) | 0.394 | 1.14 (0.44-2.99) | 0.784 |  |
| ≥30&<60 | ref | 1.50 (0.82-2.73) | 0.188 | 2.05 (1.13-3.73) | 0.019 |  |
| ≥60 | ref | 1.65 (0.75-3.62) | 0.214 | 3.05 (1.46-6.36) | 0.003 |  |
| Sex |  |  |  |  |  | 0.827 |
| Female | ref | 1.52 (0.79-2.91) | 0.206 | 2.03 (1.04-3.93) | 0.037 |  |
| Male | ref | 1.15 (0.68-1.96) | 0.598 | 1.95 (1.20-3.19) | 0.007 |  |
| BMI group/kg·m^-2^ |  |  |  |  |  | 0.681 |
| <18.5 | ref | 1.45 (0.64-3.27) | 0.370 | 1.98 (0.92-4.24) | 0.079 |  |
| ≥18.5&<24.0 | ref | 0.96 (0.54-1.72) | 0.897 | 1.94 (1.13-3.31) | 0.016 |  |
| ≥24.0 | ref | 1.97 (0.77-5.05) | 0.159 | 2.34 (0.86-6.40) | 0.097 |  |
| Smoking |  |  |  |  |  | 0.494 |
| No | ref | 1.30 (0.79-2.14) | 0.295 | 2.07 (1.28-3.34) | 0.003 |  |
| Yes | ref | 0.98 (0.46-2.07) | 0.957 | 1.83 (0.93-3.62) | 0.081 |  |
| Treatment Category |  |  |  |  |  | 0.833 |
| Initial | ref | 1.15 (0.74-1.81) | 0.533 | 1.85 (1.21-2.83) | 0.005 |  |
| Retreat | ref | 2.09 (0.77-5.66) | 0.148 | 4.54 (1.69-12.15) | 0.003 |  |
| DST |  |  |  |  |  | 0.249 |
| DS-TB | ref | 1.21 (0.75-1.95) | 0.437 | 1.30 (0.78-2.01) | 0.679 |  |
| MDR-TB | ref | 1.17 (0.68-4.98) | 0.192 | 1.97 (1.25-5.11) | 0.003 |  |
| DR_nonMDR | ref | 0.83 (0.15-4.55) | 0.831 | 3.12 (0.71-13.64) | 0.130 |  |
| Diabetes |  |  |  |  |  | 0.111 |
| No | ref | 1.00 (0.63-1.61) | 0.986 | 1.95 (1.26-3.01) | 0.003 |  |
| Yes | ref | 2.04 (0.78-5.28) | 0.144 | 2.35 (0.92-6.00) | 0.073 |  |
| HBV |  |  |  |  |  | 0.892 |
| No | ref | 1.26 (0.82-1.94) | 0.287 | 2.03 (1.36-3.05) | <0.001 |  |
| Yes | ref | 0.86 (0.21-3.60) | 0.840 | 1.99 (0.48-8.25) | 0.344 |  |
| Hypertension |  |  |  |  |  | 0.561 |
| No | ref | 1.34 (0.84-2.14) | 0.226 | 2.17 (1.39-3.40) | <0.001 |  |
| Yes | ref | 0.68 (0.29-1.63) | 0.393 | 1.15 (0.53-2.49) | 0.724 |  |
| Anemia |  |  |  |  |  | 0.582 |
| No | ref | 1.17 (0.75-1.83) | 0.496 | 2.01 (1.31-3.07) | 0.001 |  |
| Yes | ref | 1.59 (0.53-4.78) | 0.406 | 2.03 (0.70-5.89) | 0.191 |  |
| Tumor |  |  |  |  |  | 0.045 |
| No | ref | 1.01 (0.66-1.56) | 0.952 | 1.82 (1.22-2.71) | 0.003 |  |
| Yes | ref | 16.75 (1.94-144.48) | 0.010 | 11.25 (1.43-88.77) | 0.022 |  |
| EPTB |  |  |  |  |  | 0.096 |
| No | ref | 0.93 (0.54-1.59) | 0.783 | 1.87 (1.13-3.08) | 0.015 |  |
| Yes | ref | 1.84 (0.94-3.57) | 0.073 | 2.54 (1.32-4.86) | 0.005 |  |
| Cavity |  |  |  |  |  | 0.756 |
| No | ref | 1.31 (0.83-2.07) | 0.238 | 1.86 (0.73-2.72) | 0.192 |  |
| Yes | ref | 1.00 (0.38-2.68) | 0.996 | 2.07 (1.34-3.19) | 0.001 |  |

Note: CAR: C-reactive protein to albumin ratio; BMI: Body mass index; DST: Drug susceptibility testing; DS-TB: Drug-susceptible; MDR-TB: Multidrug-resistant TB; DR_nonMDR: Drug-resistant TB except MDR-TB; HBV: Hepatitis B virus; EPTB: Extrapulmonary tuberculosis.

Supplementary Table 6 Subgroup analysis of the association between CLR and unfavorable treatment outcome

| Subgroup | HR (95%*CI*) | | | | | *P* for  interaction |
| --- | --- | --- | --- | --- | --- | --- |
|  | Q1 | Q2 | *P* | Q3 | *P* |  |
| Age group/years |  |  |  |  |  | 0.005 |
| <30 | ref | 0.68 (0.25-1.81) | 0.437 | 0.78 (0.29-2.15) | 0.635 |  |
| ≥30&<60 | ref | 1.88 (1.03-3.42) | 0.040 | 2.05 (1.11-3.80) | 0.022 |  |
| ≥60 | ref | 1.61 (0.71-3.69) | 0.257 | 3.23 (1.51-6.93) | 0.003 |  |
| Sex |  |  |  |  |  | 0.777 |
| Female | ref | 1.54 (0.80-2.96) | 0.197 | 1.94 (1.00-3.79) | 0.051 |  |
| Male | ref | 1.30 (0.76-2.20) | 0.338 | 1.99 (1.20-3.28) | 0.007 |  |
| BMI group/kg·m^-2^ |  |  |  |  |  | 0.777 |
| <18.5 | ref | 1.03 (0.45-2.35) | 0.942 | 1.72 (0.83-3.59) | 0.147 |  |
| ≥18.5&<24.0 | ref | 1.15 (0.66-2.01) | 0.617 | 1.80 (1.05-3.08) | 0.032 |  |
| ≥24.0 | ref | 2.91 (1.06-7.96) | 0.038 | 3.55 (1.21-10.43) | 0.022 |  |
| Smoking |  |  |  |  |  | 0.661 |
| No | ref | 1.29 (0.79-2.12) | 0.311 | 1.95 (1.21-3.15) | 0.006 |  |
| Yes | ref | 1.22 (0.59-2.56) | 0.592 | 1.93 (0.96-3.88) | 0.065 |  |
| Treatment Category |  |  |  |  |  | 0.423 |
| Initial | ref | 1.10 (0.71-1.73) | 0.663 | 1.72 (1.12-2.63) | 0.013 |  |
| Retreat | ref | 3.14 (1.12-8.75) | 0.029 | 5.20 (1.85-14.61) | 0.002 |  |
| DST |  |  |  |  |  | 0.116 |
| DS-TB | ref | 1.20 (0.74-1.94) | 0.455 | 1.83 (0.82-2.12) | 0.787 |  |
| MDR-TB | ref | 1.73 (0.57-4.28) | 0.333 | 1.98 (1.26-4.11) | 0.003 |  |
| DR_nonMDR | ref | 2.32 (0.43-12.67) | 0.330 | 3.50 (0.63-19.60) | 0.154 |  |
| Diabetes |  |  |  |  |  | 0.126 |
| No | ref | 1.05 (0.65-1.69) | 0.842 | 1.95 (1.25-3.02) | 0.003 |  |
| Yes | ref | 1.94 (0.78-4.85) | 0.156 | 2.07 (0.82-5.21) | 0.121 |  |
| HBV |  |  |  |  |  | 0.735 |
| No | ref | 1.26 (0.82-1.93) | 0.300 | 1.97 (1.31-2.98) | 0.001 |  |
| Yes | ref | 1.56 (0.40-6.13) | 0.526 | 2.21 (0.53-9.18) | 0.276 |  |
| Hypertension |  |  |  |  |  | 0.799 |
| No | ref | 1.35 (0.85-2.15) | 0.202 | 1.93 (1.23-3.02) | 0.004 |  |
| Yes | ref | 0.87 (0.36-2.10) | 0.756 | 1.54 (0.67-3.51) | 0.309 |  |
| Anemia |  |  |  |  |  | 0.314 |
| No | ref | 1.29 (0.83-2.00) | 0.260 | 1.83 (1.20-2.81) | 0.005 |  |
| Yes | ref | 1.91 (0.57-6.43) | 0.294 | 2.81 (0.88-8.94) | 0.081 |  |
| Tumor |  |  |  |  |  | 0.121 |
| No | ref | 1.09 (0.71-1.68) | 0.680 | 1.79 (1.19-2.68) | 0.005 |  |
| Yes | ref | 7.57 (1.39-41.21) | 0.019 | 5.69 (1.02-31.82) | 0.048 |  |
| EPTB |  |  |  |  |  | 0.133 |
| No | ref | 1.06 (0.63-1.79) | 0.830 | 1.75 (1.06-2.91) | 0.030 |  |
| Yes | ref | 1.75 (0.88-3.48) | 0.109 | 2.53 (1.30-4.91) | 0.006 |  |
| Cavity |  |  |  |  |  | 0.898 |
| No | ref | 1.32 (0.83-2.09) | 0.246 | 1.34 (0.85-2.26) | 0.525 |  |
| Yes | ref | 1.02 (0.42-2.50) | 0.963 | 2.20 (1.42-3.41) | <0.001 |  |

Note: CLR: C-reactive protein to lymphocyte ratio; BMI: Body mass index; DST: Drug susceptibility testing; DS-TB: Drug-susceptible; MDR-TB: Multidrug-resistant TB; DR_nonMDR: Drug-resistant TB except MDR-TB; HBV: Hepatitis B virus; EPTB: Extrapulmonary tuberculosis.

Supplementary Table 7 Subgroup analysis of the association between dNLR and unfavorable treatment outcome

| Subgroup | HR (95%*CI*) | | | | | *P* for  interaction |
| --- | --- | --- | --- | --- | --- | --- |
|  | Q1 | Q2 | *P* | Q3 | *P* |  |
| Age group/years |  |  |  |  |  | 0.025 |
| <30 | ref | 0.55 (0.21-1.46) | 0.232 | 0.99 (0.40-2.45) | 0.975 |  |
| ≥30&<60 | ref | 1.30 (0.70-2.42) | 0.405 | 2.02 (1.13-3.60) | 0.018 |  |
| ≥60 | ref | 2.14 (1.00-4.59) | 0.051 | 3.80 (1.86-7.74) | <0.001 |  |
| Sex |  |  |  |  |  | 0.041 |
| Female | ref | 0.66 (0.33-1.32) | 0.242 | 1.58 (0.89-2.82) | 0.120 |  |
| Male | ref | 1.85 (1.08-3.17) | 0.026 | 2.88 (1.73-4.80) | <0.001 |  |
| BMI group/kg·m^-2^ |  |  |  |  |  | 0.296 |
| <18.5 | ref | 1.85 (0.71-4.83) | 0.211 | 4.51 (1.88-10.83) | <0.001 |  |
| ≥18.5&<24.0 | ref | 1.20 (0.71-2.04) | 0.493 | 1.73 (1.05-2.86) | 0.032 |  |
| ≥24.0 | ref | 1.31 (0.51-3.35) | 0.580 | 2.09 (0.87-5.06) | 0.101 |  |
| Smoking |  |  |  |  |  | 0.544 |
| No | ref | 1.17 (0.71-1.93) | 0.549 | 2.24 (1.43-3.51) | <0.001 |  |
| Yes | ref | 1.49 (0.74-3.00) | 0.270 | 2.37 (1.20-4.68) | 0.013 |  |
| Treatment Category |  |  |  |  |  | 0.092 |
| Initial | ref | 1.03 (0.66-1.61) | 0.911 | 1.92 (1.29-2.87) | 0.001 |  |
| Retreat | ref | 4.11 (1.36-12.45) | 0.012 | 5.29 (1.78-15.70) | 0.003 |  |
| DST |  |  |  |  |  | 0.538 |
| DS-TB | ref | 1.19 (0.76-1.88) | 0.446 | 1.36 (0.85-1.81) | 0.147 |  |
| MDR-TB | ref | 0.99 (0.32-3.03) | 0.988 | 1.89 (1.25-3.87) | 0.003 |  |
| DR_nonMDR | ref | 1.43 (0.21-9.48) | 0.713 | 5.69 (1.06-30.53) | 0.043 |  |
| Diabetes |  |  |  |  |  | 0.270 |
| No | ref | 1.09 (0.67-1.77) | 0.727 | 2.35 (1.53-3.62) | <0.001 |  |
| Yes | ref | 1.51 (0.70-3.24) | 0.291 | 1.70 (0.82-3.50) | 0.152 |  |
| HBV |  |  |  |  |  | 0.037 |
| No | ref | 1.22 (0.79-1.88) | 0.378 | 2.37 (1.60-3.51) | <0.001 |  |
| Yes | ref | 1.68 (0.48-5.92) | 0.416 | 1.03 (0.25-4.28) | 0.964 |  |
| Hypertension |  |  |  |  |  | 0.196 |
| No | ref | 1.11 (0.71-1.73) | 0.650 | 1.78 (1.18-2.67) | 0.006 |  |
| Yes | ref | 1.89 (0.66-5.38) | 0.236 | 4.42 (1.65-11.86) | 0.003 |  |
| Anemia |  |  |  |  |  | 0.059 |
| No | ref | 1.31 (0.85-2.03) | 0.220 | 1.91 (1.27-2.88) | 0.002 |  |
| Yes | ref | 1.19 (0.38-3.70) | 0.765 | 3.64 (1.32-10.08) | 0.013 |  |
| Tumor |  |  |  |  |  | 0.874 |
| No | ref | 1.21 (0.79-1.86) | 0.383 | 2.14 (1.45-3.17) | <0.001 |  |
| Yes | ref | 2.00 (0.51-7.81) | 0.318 | 2.29 (0.66-7.93) | 0.190 |  |
| EPTB |  |  |  |  |  | 0.969 |
| No | ref | 1.30 (0.75-2.23) | 0.351 | 2.43 (1.47-4.03) | <0.001 |  |
| Yes | ref | 1.11 (0.60-2.05) | 0.739 | 1.84 (1.05-3.21) | 0.032 |  |
| Cavity |  |  |  |  |  | 0.776 |
| No | ref | 1.26 (0.80-2.00) | 0.324 | 1.71 (0.77-2.77) | 0.185 |  |
| Yes | ref | 1.34 (0.56-3.18) | 0.510 | 2.36 (1.55-3.60) | <0.001 |  |

Note: dNLR: Derived neutrophil to lymphocyte ratio; BMI: Body mass index; DST: Drug susceptibility testing; DS-TB: Drug-susceptible; MDR-TB: Multidrug-resistant TB; DR_nonMDR: Drug-resistant TB except MDR-TB; HBV: Hepatitis B virus; EPTB: Extrapulmonary tuberculosis.

Supplementary Table 8 Subgroup analysis of the association between ELR and unfavorable treatment outcome

| Subgroup | HR (95%*CI*) | | | | | *P* for  interaction |
| --- | --- | --- | --- | --- | --- | --- |
|  | Q1 | Q2 | *P* | Q3 | *P* |  |
| Age group/years |  |  |  |  |  | 0.237 |
| <30 | ref | 0.54 (0.23-1.30) | 0.170 | 0.55 (0.19-1.60) | 0.272 |  |
| ≥30&<60 | ref | 0.90 (0.52-1.56) | 0.705 | 1.35 (0.82-2.23) | 0.241 |  |
| ≥60 | ref | 1.01 (0.60-1.73) | 0.958 | 0.89 (0.56-1.42) | 0.631 |  |
| Sex |  |  |  |  |  | 0.867 |
| Female | ref | 0.91 (0.49-1.68) | 0.756 | 1.11 (0.60-2.03) | 0.743 |  |
| Male | ref | 0.82 (0.54-1.25) | 0.355 | 0.88 (0.61-1.27) | 0.493 |  |
| BMI group/kg·m^-2^ |  |  |  |  |  | 0.056 |
| <18.5 | ref | 0.51 (0.28-0.95) | 0.033 | 0.66 (0.40-1.08) | 0.098 |  |
| ≥18.5&<24.0 | ref | 1.15 (0.69-1.92) | 0.587 | 1.43 (0.89-2.28) | 0.141 |  |
| ≥24.0 | ref | 0.77 (0.34-1.74) | 0.527 | 0.59 (0.23-1.55) | 0.285 |  |
| Smoking |  |  |  |  |  | 0.121 |
| No | ref | 0.79 (0.51-1.21) | 0.280 | 1.05 (0.72-1.54) | 0.800 |  |
| Yes | ref | 1.00 (0.56-1.78) | 0.987 | 0.71 (0.41-1.24) | 0.226 |  |
| Treatment Category |  |  |  |  |  | 0.479 |
| Initial | ref | 0.82 (0.56-1.21) | 0.319 | 0.84 (0.59-1.19) | 0.323 |  |
| Retreat | ref | 1.15 (0.50-2.63) | 0.747 | 1.43 (0.68-3.00) | 0.343 |  |
| DST |  |  |  |  |  | 0.372 |
| DS-TB | ref | 0.88 (0.60-1.31) | 0.540 | 0.99 (0.81-1.70) | 0.905 |  |
| MDR-TB | ref | 0.84 (0.30-2.35) | 0.740 | 1.02 (0.32-1.95) | 0.983 |  |
| DR_nonMDR | ref | 0.60 (0.16-2.24) | 0.445 | 0.58 (0.14-2.40) | 0.450 |  |
| Diabetes |  |  |  |  |  | 0.118 |
| No | ref | 0.69 (0.45-1.05) | 0.086 | 0.89 (0.62-1.30) | 0.554 |  |
| Yes | ref | 1.39 (0.75-2.57) | 0.295 | 1.07 (0.58-1.96) | 0.829 |  |
| HBV |  |  |  |  |  | 0.335 |
| No | ref | 0.78 (0.54-1.12) | 0.184 | 0.87 (0.63-1.21) | 0.410 |  |
| Yes | ref | 2.14 (0.57-8.03) | 0.259 | 1.53 (0.41-5.75) | 0.529 |  |
| Hypertension |  |  |  |  |  | 0.846 |
| No | ref | 0.83 (0.56-1.24) | 0.363 | 0.93 (0.65-1.33) | 0.680 |  |
| Yes | ref | 1.08 (0.51-2.25) | 0.848 | 0.97 (0.51-1.88) | 0.937 |  |
| Anemia |  |  |  |  |  | 0.501 |
| No | ref | 0.79 (0.53-1.17) | 0.237 | 0.94 (0.65-1.36) | 0.748 |  |
| Yes | ref | 1.24 (0.61-2.56) | 0.552 | 1.07 (0.58-1.96) | 0.825 |  |
| Tumor |  |  |  |  |  | 0.392 |
| No | ref | 0.89 (0.62-1.29) | 0.544 | 1.03 (0.73-1.44) | 0.875 |  |
| Yes | ref | 0.69 (0.23-2.06) | 0.507 | 0.58 (0.24-1.43) | 0.235 |  |
| EPTB |  |  |  |  |  | 0.585 |
| No | ref | 0.79 (0.50-1.25) | 0.315 | 0.84 (0.54-1.28) | 0.412 |  |
| Yes | ref | 0.88 (0.52-1.50) | 0.649 | 1.10 (0.69-1.76) | 0.689 |  |
| Cavity |  |  |  |  |  | 0.621 |
| No | ref | 0.96 (0.63-1.44) | 0.827 | 1.03 (0.71-1.50) | 0.879 |  |
| Yes | ref | 0.72 (0.37-1.40) | 0.339 | 0.74 (0.41-1.34) | 0.323 |  |

Note: ELR: Eosinophil to lymphocyte ratio; BMI: Body mass index; DST: Drug susceptibility testing; DS-TB: Drug-susceptible; MDR-TB: Multidrug-resistant TB; DR_nonMDR: Drug-resistant TB except MDR-TB; HBV: Hepatitis B virus; EPTB: Extrapulmonary tuberculosis.

Supplementary Table 9 Subgroup analysis of the association between EMR and unfavorable treatment outcome

| Subgroup | HR (95%*CI*) | | | | | *P* for  interaction |
| --- | --- | --- | --- | --- | --- | --- |
|  | Q1 | Q2 | *P* | Q3 | *P* |  |
| Age group/years |  |  |  |  |  | 0.116 |
| <30 | ref | 0.73 (0.30-1.76) | 0.481 | 0.70 (0.28-1.77) | 0.454 |  |
| ≥30&<60 | ref | 1.63 (0.99-2.66) | 0.052 | 0.84 (0.48-1.47) | 0.540 |  |
| ≥60 | ref | 0.70 (0.43-1.14) | 0.149 | 0.69 (0.43-1.11) | 0.123 |  |
| Sex |  |  |  |  |  | 0.157 |
| Female | ref | 0.74 (0.39-1.39) | 0.348 | 0.98 (0.54-1.77) | 0.946 |  |
| Male | ref | 1.04 (0.72-1.49) | 0.848 | 0.64 (0.42-0.96) | 0.030 |  |
| BMI group/kg·m^-2^ |  |  |  |  |  | 0.036 |
| <18.5 | ref | 0.56 (0.32-1.00) | 0.048 | 0.65 (0.38-1.10) | 0.110 |  |
| ≥18.5&<24.0 | ref | 1.47 (0.93-2.34) | 0.103 | 1.05 (0.64-1.73) | 0.840 |  |
| ≥24.0 | ref | 0.70 (0.32-1.56) | 0.387 | 0.35 (0.14-0.90) | 0.029 |  |
| Smoking |  |  |  |  |  | 0.374 |
| No | ref | 1.02 (0.69-1.50) | 0.936 | 0.89 (0.59-1.34) | 0.574 |  |
| Yes | ref | 0.87 (0.51-1.48) | 0.600 | 0.49 (0.27-0.88) | 0.017 |  |
| Treatment Category |  |  |  |  |  | 0.523 |
| Initial | ref | 0.90 (0.63-1.28) | 0.561 | 0.70 (0.48-1.01) | 0.059 |  |
| Retreat | ref | 1.35 (0.67-2.70) | 0.403 | 0.80 (0.37-1.71) | 0.568 |  |
| DST |  |  |  |  |  | 0.728 |
| DS-TB | ref | 1.01 (0.71-1.42) | 0.973 | 0.76 (0.52-1.10) | 0.147 |  |
| MDR-TB | ref | 0.55 (0.19-1.57) | 0.260 | 0.88 (0.31-2.48) | 0.804 |  |
| DR_nonMDR | ref | 1.15 (0.38-3.55) | 0.802 | 0.53 (0.12-2.28) | 0.395 |  |
| Diabetes |  |  |  |  |  | 0.914 |
| No | ref | 0.90 (0.62-1.31) | 0.596 | 0.71 (0.48-1.07) | 0.101 |  |
| Yes | ref | 0.99 (0.55-1.76) | 0.964 | 0.82 (0.44-1.50) | 0.514 |  |
| HBV |  |  |  |  |  | 0.119 |
| No | ref | 0.87 (0.63-1.20) | 0.395 | 0.65 (0.46-0.92) | 0.016 |  |
| Yes | ref | 1.31 (0.37-4.72) | 0.677 | 1.99 (0.51-7.75) | 0.320 |  |
| Hypertension |  |  |  |  |  | 0.689 |
| No | ref | 1.01 (0.71-1.44) | 0.957 | 0.78 (0.53-1.14) | 0.198 |  |
| Yes | ref | 0.76 (0.39-1.49) | 0.423 | 0.62 (0.31-1.23) | 0.171 |  |
| Anemia |  |  |  |  |  | 0.168 |
| No | ref | 0.99 (0.69-1.43) | 0.972 | 0.86 (0.58-1.26) | 0.438 |  |
| Yes | ref | 1.07 (0.58-1.98) | 0.829 | 0.50 (0.24-1.04) | 0.064 |  |
| Tumor |  |  |  |  |  | 0.047 |
| No | ref | 1.05 (0.75-1.47) | 0.775 | 0.85 (0.60-1.21) | 0.358 |  |
| Yes | ref | 0.50 (0.19-1.28) | 0.147 | 0.15 (0.04-0.59) | 0.007 |  |
| EPTB |  |  |  |  |  | 0.186 |
| No | ref | 0.74 (0.49-1.14) | 0.176 | 0.57 (0.36-0.89) | 0.014 |  |
| Yes | ref | 1.46 (0.92-2.33) | 0.111 | 1.06 (0.64-1.74) | 0.829 |  |
| Cavity |  |  |  |  |  | 0.337 |
| No | ref | 0.88 (0.61-1.29) | 0.519 | 0.77 (0.53-1.14) | 0.191 |  |
| Yes | ref | 1.26 (0.71-2.22) | 0.432 | 0.57 (0.28-1.14) | 0.110 |  |

Note: EMR: Eosinophil to monocyte ratio; BMI: Body mass index; DST: Drug susceptibility testing; DS-TB: Drug-susceptible; MDR-TB: Multidrug-resistant TB; DR_nonMDR: Drug-resistant TB except MDR-TB; HBV: Hepatitis B virus; EPTB: Extrapulmonary tuberculosis.

Supplementary Table 10 Subgroup analysis of the association between ENR and unfavorable treatment outcome

| Subgroup | HR (95%*CI*) | | | | | *P* for  interaction |
| --- | --- | --- | --- | --- | --- | --- |
|  | Q1 | Q2 | *P* | Q3 | *P* |  |
| Age group/years |  |  |  |  |  | 0.225 |
| <30 | ref | 0.49 (0.19-1.26) | 0.139 | 0.70 (0.29-1.69) | 0.426 |  |
| ≥30&<60 | ref | 1.28 (0.79-2.08) | 0.320 | 0.88 (0.51-1.52) | 0.645 |  |
| ≥60 | ref | 0.63 (0.39-1.03) | 0.064 | 0.67 (0.43-1.07) | 0.095 |  |
| Sex |  |  |  |  |  | 0.340 |
| Female | ref | 0.64 (0.34-1.21) | 0.171 | 0.87 (0.49-1.56) | 0.650 |  |
| Male | ref | 0.88 (0.61-1.27) | 0.496 | 0.65 (0.44-0.97) | 0.035 |  |
| BMI group/kg·m^-2^ |  |  |  |  |  | 0.009 |
| <18.5 | ref | 0.37 (0.20-0.68) | 0.001 | 0.51 (0.29-0.87) | 0.015 |  |
| ≥18.5&<24.0 | ref | 1.43 (0.90-2.28) | 0.133 | 1.09 (0.66-1.80) | 0.730 |  |
| ≥24.0 | ref | 0.61 (0.26-1.44) | 0.257 | 0.58 (0.24-1.39) | 0.220 |  |
| Smoking |  |  |  |  |  | 0.351 |
| No | ref | 0.95 (0.65-1.41) | 0.814 | 0.80 (0.53-1.21) | 0.285 |  |
| Yes | ref | 0.53 (0.29-0.95) | 0.033 | 0.57 (0.33-0.98) | 0.044 |  |
| Treatment Category |  |  |  |  |  | 0.455 |
| Initial | ref | 0.75 (0.52-1.09) | 0.128 | 0.73 (0.51-1.05) | 0.086 |  |
| Retreat | ref | 0.95 (0.48-1.86) | 0.880 | 0.58 (0.26-1.27) | 0.174 |  |
| DST |  |  |  |  |  | 0.282 |
| DS-TB | ref | 0.92 (0.65-1.31) | 0.652 | 0.79 (0.55-1.13) | 0.193 |  |
| MDR-TB | ref | 0.59 (0.21-1.63) | 0.306 | 0.89 (0.32-2.48) | 0.818 |  |
| DR_nonMDR | ref | 0.19 (0.05-0.78) | 0.021 | 0.33 (0.08-1.35) | 0.123 |  |
| Diabetes |  |  |  |  |  | 0.298 |
| No | ref | 0.68 (0.46-1.00) | 0.047 | 0.64 (0.43-0.94) | 0.024 |  |
| Yes | ref | 1.13 (0.63-2.00) | 0.688 | 1.04 (0.56-1.91) | 0.913 |  |
| HBV |  |  |  |  |  | 0.156 |
| No | ref | 0.74 (0.53-1.02) | 0.070 | 0.65 (0.46-0.91) | 0.013 |  |
| Yes | ref | 2.49 (0.68-9.07) | 0.166 | 1.64 (0.42-6.46) | 0.476 |  |
| Hypertension |  |  |  |  |  | 0.790 |
| No | ref | 0.84 (0.58-1.21) | 0.337 | 0.76 (0.52-1.10) | 0.144 |  |
| Yes | ref | 0.67 (0.35-1.28) | 0.222 | 0.62 (0.31-1.24) | 0.178 |  |
| Anemia |  |  |  |  |  | 0.148 |
| No | ref | 0.82 (0.56-1.18) | 0.282 | 0.84 (0.58-1.22) | 0.357 |  |
| Yes | ref | 0.88 (0.47-1.66) | 0.699 | 0.50 (0.24-1.03) | 0.061 |  |
| Tumor |  |  |  |  |  | 0.258 |
| No | ref | 0.81 (0.57-1.14) | 0.224 | 0.79 (0.56-1.12) | 0.183 |  |
| Yes | ref | 0.69 (0.27-1.75) | 0.435 | 0.37 (0.12-1.19) | 0.096 |  |
| EPTB |  |  |  |  |  | 0.868 |
| No | ref | 0.72 (0.46-1.11) | 0.135 | 0.69 (0.44-1.07) | 0.100 |  |
| Yes | ref | 0.97 (0.61-1.54) | 0.896 | 0.78 (0.48-1.28) | 0.325 |  |
| Cavity |  |  |  |  |  | 0.807 |
| No | ref | 0.81 (0.56-1.19) | 0.282 | 0.74 (0.51-1.08) | 0.124 |  |
| Yes | ref | 0.94 (0.52-1.72) | 0.845 | 0.62 (0.32-1.20) | 0.156 |  |

Note: ENR: Eosinophil to neutrophil ratio; BMI: Body mass index; DST: Drug susceptibility testing; DS-TB: Drug-susceptible; MDR-TB: Multidrug-resistant TB; DR_nonMDR: Drug-resistant TB except MDR-TB; HBV: Hepatitis B virus; EPTB: Extrapulmonary tuberculosis.

Supplementary Table 11 Subgroup analysis of the association between MLR and unfavorable treatment outcome

| Subgroup | HR (95%*CI*) | | | | | *P* for  interaction |
| --- | --- | --- | --- | --- | --- | --- |
|  | Q1 | Q2 | *P* | Q3 | *P* |  |
| Age group/years |  |  |  |  |  | 0.013 |
| <30 | ref | 0.82 (0.34-2.00) | 0.667 | 0.77 (0.28-2.08) | 0.605 |  |
| ≥30&<60 | ref | 1.27 (0.73-2.22) | 0.402 | 1.38 (0.78-2.44) | 0.261 |  |
| ≥60 | ref | 0.51 (0.24-1.08) | 0.080 | 1.57 (0.86-2.88) | 0.146 |  |
| Sex |  |  |  |  |  | 0.622 |
| Female | ref | 0.90 (0.48-1.70) | 0.753 | 1.22 (0.65-2.27) | 0.537 |  |
| Male | ref | 0.79 (0.48-1.31) | 0.362 | 1.45 (0.92-2.30) | 0.110 |  |
| BMI group/kg·m^-2^ |  |  |  |  |  | 0.555 |
| <18.5 | ref | 1.31 (0.55-3.12) | 0.539 | 2.33 (1.05-5.15) | 0.038 |  |
| ≥18.5&<24.0 | ref | 0.66 (0.39-1.14) | 0.137 | 1.14 (0.69-1.86) | 0.615 |  |
| ≥24.0 | ref | 0.99 (0.42-2.36) | 0.982 | 1.97 (0.78-4.97) | 0.154 |  |
| Smoking |  |  |  |  |  | 0.643 |
| No | ref | 0.86 (0.54-1.38) | 0.530 | 1.27 (0.82-1.97) | 0.287 |  |
| Yes | ref | 0.68 (0.32-1.45) | 0.318 | 1.67 (0.88-3.19) | 0.119 |  |
| Treatment Category |  |  |  |  |  | 0.696 |
| Initial | ref | 0.73 (0.47-1.15) | 0.178 | 1.34 (0.89-2.01) | 0.159 |  |
| Retreat | ref | 1.03 (0.44-2.40) | 0.950 | 1.74 (0.78-3.90) | 0.179 |  |
| DST |  |  |  |  |  | 0.010 |
| DS-TB | ref | 0.82 (0.52-1.29) | 0.392 | 1.42 (0.94-2.15) | 0.094 |  |
| MDR-TB | ref | 0.30 (0.08-1.13) | 0.075 | 1.06 (0.36-3.06) | 0.919 |  |
| DR_nonMDR | ref | 2.89 (0.58-14.47) | 0.196 | 2.31 (0.39-13.87) | 0.358 |  |
| Diabetes |  |  |  |  |  | 0.930 |
| No | ref | 0.85 (0.53-1.37) | 0.507 | 1.44 (0.93-2.21) | 0.099 |  |
| Yes | ref | 0.69 (0.34-1.42) | 0.317 | 1.29 (0.65-2.57) | 0.462 |  |
| HBV |  |  |  |  |  | 0.253 |
| No | ref | 0.85 (0.56-1.30) | 0.453 | 1.52 (1.03-2.23) | 0.034 |  |
| Yes | ref | 0.50 (0.14-1.77) | 0.282 | 0.57 (0.14-2.24) | 0.416 |  |
| Hypertension |  |  |  |  |  | 0.434 |
| No | ref | 0.89 (0.57-1.38) | 0.599 | 1.41 (0.94-2.13) | 0.100 |  |
| Yes | ref | 0.47 (0.19-1.16) | 0.101 | 1.10 (0.50-2.44) | 0.814 |  |
| Anemia |  |  |  |  |  | 0.321 |
| No | ref | 0.87 (0.57-1.33) | 0.529 | 1.33 (0.89-1.97) | 0.161 |  |
| Yes | ref | 0.69 (0.22-2.17) | 0.528 | 1.53 (0.55-4.29) | 0.419 |  |
| Tumor |  |  |  |  |  | 0.328 |
| No | ref | 0.73 (0.48-1.10) | 0.133 | 1.31 (0.90-1.91) | 0.156 |  |
| Yes | ref | 3.85 (0.74-20.12) | 0.110 | 4.65 (0.92-23.60) | 0.064 |  |
| EPTB |  |  |  |  |  | 0.729 |
| No | ref | 1.01 (0.60-1.71) | 0.965 | 1.83 (1.12-3.00) | 0.016 |  |
| Yes | ref | 0.61 (0.33-1.12) | 0.109 | 0.95 (0.55-1.64) | 0.858 |  |
| Cavity |  |  |  |  |  | 0.437 |
| No | ref | 0.98 (0.62-1.55) | 0.937 | 0.86 (0.43-1.74) | 0.680 |  |
| Yes | ref | 0.47 (0.21-1.04) | 0.062 | 1.66 (1.08-2.54) | 0.019 |  |

Note: MLR: Monocyte to lymphocyte ratio; BMI: Body mass index; DST: Drug susceptibility testing; DS-TB: Drug-susceptible; MDR-TB: Multidrug-resistant TB; DR_nonMDR: Drug-resistant TB except MDR-TB; HBV: Hepatitis B virus; EPTB: Extrapulmonary tuberculosis.

Supplementary Table 12 Subgroup analysis of the association between NLR and unfavorable treatment outcome

| Subgroup | HR (95%*CI*) | | | | | *P* for  interaction |
| --- | --- | --- | --- | --- | --- | --- |
|  | Q1 | Q2 | *P* | Q3 | *P* |  |
| Age group/years |  |  |  |  |  | 0.029 |
| <30 | ref | 0.93 (0.39-2.24) | 0.873 | 0.87 (0.32-2.38) | 0.790 |  |
| ≥30&<60 | ref | 1.01 (0.55-1.86) | 0.978 | 1.87 (1.05-3.30) | 0.032 |  |
| ≥60 | ref | 0.99 (0.44-2.23) | 0.972 | 2.99 (1.51-5.92) | 0.002 |  |
| Sex |  |  |  |  |  | 0.379 |
| Female | ref | 0.74 (0.37-1.49) | 0.405 | 1.75 (0.97-3.17) | 0.064 |  |
| Male | ref | 1.17 (0.68-2.01) | 0.582 | 2.32 (1.43-3.77) | <0.001 |  |
| BMI group/kg·m^-2^ |  |  |  |  |  | 0.073 |
| <18.5 | ref | 1.39 (0.47-4.14) | 0.554 | 5.00 (1.94-12.87) | <0.001 |  |
| ≥18.5&<24.0 | ref | 0.96 (0.56-1.64) | 0.883 | 1.47 (0.89-2.41) | 0.133 |  |
| ≥24.0 | ref | 0.83 (0.32-2.16) | 0.697 | 2.06 (0.88-4.82) | 0.097 |  |
| Smoking |  |  |  |  |  | 0.628 |
| No | ref | 0.92 (0.55-1.55) | 0.755 | 2.05 (1.31-3.20) | 0.002 |  |
| Yes | ref | 1.15 (0.55-2.41) | 0.707 | 2.18 (1.10-4.30) | 0.025 |  |
| Treatment Category |  |  |  |  |  | 0.090 |
| Initial | ref | 0.81 (0.50-1.30) | 0.377 | 1.96 (1.31-2.92) | 0.001 |  |
| Retreat | ref | 2.33 (0.86-6.29) | 0.096 | 3.08 (1.20-7.90) | 0.019 |  |
| DST |  |  |  |  |  | 0.082 |
| DS-TB | ref | 0.74 (0.45-1.19) | 0.213 | 1.07 (0.54-1.34) | 0.362 |  |
| MDR-TB | ref | 1.68 (0.59-4.76) | 0.330 | 1.77 (1.18-5.67) | 0.006 |  |
| DR_nonMDR | ref | 2.40 (0.42-13.87) | 0.327 | 5.97 (1.04-34.16) | 0.045 |  |
| Diabetes |  |  |  |  |  | 0.145 |
| No | ref | 0.80 (0.48-1.34) | 0.403 | 2.13 (1.39-3.26) | <0.001 |  |
| Yes | ref | 1.24 (0.57-2.70) | 0.596 | 1.65 (0.79-3.43) | 0.181 |  |
| HBV |  |  |  |  |  | 0.227 |
| No | ref | 1.00 (0.64-1.57) | 0.993 | 2.21 (1.49-3.28) | <0.001 |  |
| Yes | ref | 0.99 (0.28-3.49) | 0.984 | 1.05 (0.29-3.83) | 0.940 |  |
| Hypertension |  |  |  |  |  | 0.448 |
| No | ref | 0.91 (0.57-1.44) | 0.686 | 1.73 (1.15-2.60) | 0.008 |  |
| Yes | ref | 1.12 (0.39-3.20) | 0.837 | 3.22 (1.28-8.09) | 0.013 |  |
| Anemia |  |  |  |  |  | 0.059 |
| No | ref | 1.05 (0.67-1.64) | 0.826 | 1.83 (1.22-2.75) | 0.004 |  |
| Yes | ref | 0.66 (0.18-2.40) | 0.528 | 3.02 (1.08-8.47) | 0.035 |  |
| Tumor |  |  |  |  |  | 0.408 |
| No | ref | 0.90 (0.58-1.40) | 0.635 | 2.01 (1.37-2.96) | <0.001 |  |
| Yes | ref | 2.01 (0.44-9.23) | 0.370 | 2.57 (0.63-10.41) | 0.186 |  |
| EPTB |  |  |  |  |  | 0.698 |
| No | ref | 0.91 (0.52-1.58) | 0.738 | 2.06 (1.27-3.34) | 0.003 |  |
| Yes | ref | 0.98 (0.51-1.90) | 0.964 | 1.96 (1.09-3.54) | 0.025 |  |
| Cavity |  |  |  |  |  | 0.900 |
| No | ref | 1.01 (0.63-1.64) | 0.954 | 1.49 (0.67-2.32) | 0.330 |  |
| Yes | ref | 0.88 (0.36-2.16) | 0.781 | 2.21 (1.46-3.35) | <0.001 |  |

Note: NLR: Neutrophil to lymphocyte ratio; BMI: Body mass index; DST: Drug susceptibility testing; DS-TB: Drug-susceptible; MDR-TB: Multidrug-resistant TB; DR_nonMDR: Drug-resistant TB except MDR-TB; HBV: Hepatitis B virus; EPTB: Extrapulmonary tuberculosis.

Supplementary Table 13 Subgroup analysis of the association between PLR and unfavorable treatment outcome

| Subgroup | HR (95%*CI*) | | | | | *P* for  interaction |
| --- | --- | --- | --- | --- | --- | --- |
|  | Q1 | Q2 | *P* | Q3 | *P* |  |
| Age group/years |  |  |  |  |  | 0.011 |
| <30 | ref | 1.62 (0.69-3.84) | 0.270 | 0.63 (0.20-2.03) | 0.442 |  |
| ≥30&<60 | ref | 1.62 (0.97-2.70) | 0.065 | 0.88 (0.49-1.60) | 0.681 |  |
| ≥60 | ref | 0.86 (0.48-1.53) | 0.606 | 1.25 (0.75-2.06) | 0.390 |  |
| Sex |  |  |  |  |  | 0.011 |
| Female | ref | 1.10 (0.61-1.98) | 0.756 | 0.52 (0.25-1.06) | 0.070 |  |
| Male | ref | 1.21 (0.78-1.86) | 0.392 | 1.32 (0.86-2.01) | 0.203 |  |
| BMI group/kg·m^-2^ |  |  |  |  |  | 0.027 |
| <18.5 | ref | 2.22 (0.99-5.01) | 0.054 | 2.12 (0.97-4.62) | 0.060 |  |
| ≥18.5&<24.0 | ref | 1.24 (0.78-1.96) | 0.365 | 0.85 (0.51-1.42) | 0.539 |  |
| ≥24.0 | ref | 0.67 (0.26-1.69) | 0.392 | 1.06 (0.41-2.75) | 0.897 |  |
| Smoking |  |  |  |  |  | 0.329 |
| No | ref | 0.93 (0.61-1.41) | 0.728 | 0.81 (0.53-1.25) | 0.343 |  |
| Yes | ref | 1.69 (0.90-3.16) | 0.102 | 1.79 (0.94-3.39) | 0.076 |  |
| Treatment Category |  |  |  |  |  | 0.152 |
| Initial | ref | 1.30 (0.88-1.92) | 0.180 | 0.96 (0.64-1.45) | 0.857 |  |
| Retreat | ref | 0.90 (0.40-2.03) | 0.795 | 1.64 (0.78-3.44) | 0.189 |  |
| DST |  |  |  |  |  | 0.643 |
| DS-TB | ref | 1.03 (0.70-1.53) | 0.233 | 1.27 (0.86-1.89) | 0.867 |  |
| MDR-TB | ref | 0.83 (0.23-2.98) | 0.657 | 1.25 (0.47-3.37) | 0.774 |  |
| DR_nonMDR | ref | 1.43 (0.39-5.28) | 0.593 | 2.20 (0.51-9.54) | 0.294 |  |
| Diabetes |  |  |  |  |  | 0.615 |
| No | ref | 1.13 (0.73-1.74) | 0.574 | 1.13 (0.74-1.71) | 0.573 |  |
| Yes | ref | 1.28 (0.71-2.32) | 0.417 | 0.82 (0.41-1.61) | 0.558 |  |
| HBV |  |  |  |  |  | 0.097 |
| No | ref | 1.12 (0.78-1.61) | 0.547 | 1.10 (0.76-1.58) | 0.619 |  |
| Yes | ref | 2.62 (0.85-8.03) | 0.093 | 0.51 (0.10-2.56) | 0.416 |  |
| Hypertension |  |  |  |  |  | 0.725 |
| No | ref | 1.12 (0.75-1.67) | 0.580 | 1.04 (0.70-1.55) | 0.841 |  |
| Yes | ref | 1.34 (0.66-2.72) | 0.418 | 0.91 (0.41-2.01) | 0.817 |  |
| Anemia |  |  |  |  |  | 0.399 |
| No | ref | 1.16 (0.78-1.72) | 0.465 | 1.23 (0.83-1.82) | 0.309 |  |
| Yes | ref | 1.63 (0.73-3.61) | 0.231 | 0.69 (0.31-1.52) | 0.352 |  |
| Tumor |  |  |  |  |  | 0.268 |
| No | ref | 1.26 (0.88-1.82) | 0.213 | 1.09 (0.74-1.59) | 0.660 |  |
| Yes | ref | 0.65 (0.19-2.24) | 0.493 | 0.78 (0.28-2.17) | 0.637 |  |
| EPTB |  |  |  |  |  | 0.600 |
| No | ref | 1.11 (0.71-1.74) | 0.657 | 1.21 (0.75-1.93) | 0.434 |  |
| Yes | ref | 1.41 (0.81-2.48) | 0.227 | 0.96 (0.55-1.69) | 0.897 |  |
| Cavity |  |  |  |  |  | 0.368 |
| No | ref | 1.34 (0.90-1.99) | 0.150 | 1.06 (0.70-1.60) | 0.800 |  |
| Yes | ref | 0.90 (0.44-1.83) | 0.761 | 0.99 (0.50-1.95) | 0.966 |  |

Note: PLR: Platelet to lymphocyte ratio; BMI: Body mass index; DST: Drug susceptibility testing; DS-TB: Drug-susceptible; MDR-TB: Multidrug-resistant TB; DR_nonMDR: Drug-resistant TB except MDR-TB; HBV: Hepatitis B virus; EPTB: Extrapulmonary tuberculosis.

Supplementary Table 14 Subgroup analysis of the association between PNI and unfavorable treatment outcome

| Subgroup | HR (95%*CI*) | | | | | *P* for  interaction |
| --- | --- | --- | --- | --- | --- | --- |
|  | Q1 | Q2 | *P* | Q3 | *P* |  |
| Age group/years |  |  |  |  |  | 0.053 |
| <30 | ref | 1.04 (0.44-2.47) | 0.925 | 0.99 (0.37-2.67) | 0.991 |  |
| ≥30&<60 | ref | 0.72 (0.43-1.22) | 0.223 | 0.68 (0.40-1.15) | 0.147 |  |
| ≥60 | ref | 1.02 (0.55-1.88) | 0.952 | 1.67 (0.99-2.80) | 0.055 |  |
| Sex |  |  |  |  |  | 0.179 |
| Female | ref | 0.89 (0.49-1.62) | 0.697 | 0.86 (0.46-1.60) | 0.628 |  |
| Male | ref | 0.76 (0.49-1.19) | 0.236 | 1.21 (0.82-1.78) | 0.330 |  |
| BMI group/kg·m^-2^ |  |  |  |  |  | 0.306 |
| <18.5 | ref | 0.91 (0.45-1.86) | 0.803 | 1.62 (0.88-2.98) | 0.118 |  |
| ≥18.5&<24.0 | ref | 0.72 (0.44-1.18) | 0.188 | 0.91 (0.58-1.43) | 0.687 |  |
| ≥24.0 | ref | 1.41 (0.62-3.23) | 0.411 | 1.00 (0.38-2.64) | 0.992 |  |
| Smoking |  |  |  |  |  | 0.745 |
| No | ref | 0.90 (0.58-1.40) | 0.637 | 1.10 (0.74-1.66) | 0.634 |  |
| Yes | ref | 0.64 (0.34-1.21) | 0.173 | 1.23 (0.71-2.15) | 0.463 |  |
| Treatment Category |  |  |  |  |  | 0.931 |
| Initial | ref | 0.81 (0.54-1.21) | 0.307 | 1.11 (0.77-1.61) | 0.567 |  |
| Retreat | ref | 0.89 (0.39-2.02) | 0.780 | 1.09 (0.54-2.17) | 0.816 |  |
| DST |  |  |  |  |  | 0.145 |
| DS-TB | ref | 0.76 (0.50-1.14) | 0.184 | 1.16 (0.81-1.66) | 0.415 |  |
| MDR-TB | ref | 1.91 (0.65-5.62) | 0.239 | 1.78 (0.60-5.30) | 0.302 |  |
| DR_nonMDR | ref | 1.35 (0.42-4.32) | 0.614 | 0.33 (0.07-1.43) | 0.137 |  |
| Diabetes |  |  |  |  |  | 0.279 |
| No | ref | 0.99 (0.64-1.54) | 0.972 | 1.36 (0.91-2.02) | 0.134 |  |
| Yes | ref | 0.61 (0.32-1.15) | 0.129 | 0.74 (0.41-1.33) | 0.312 |  |
| HBV |  |  |  |  |  | 0.246 |
| No | ref | 0.82 (0.56-1.20) | 0.315 | 1.18 (0.84-1.66) | 0.335 |  |
| Yes | ref | 0.78 (0.22-2.74) | 0.699 | 0.50 (0.15-1.69) | 0.265 |  |
| Hypertension |  |  |  |  |  | 0.557 |
| No | ref | 0.75 (0.50-1.13) | 0.167 | 0.99 (0.68-1.44) | 0.956 |  |
| Yes | ref | 1.03 (0.47-2.23) | 0.946 | 1.37 (0.69-2.72) | 0.364 |  |
| Anemia |  |  |  |  |  | 0.562 |
| No | ref | 0.84 (0.57-1.26) | 0.408 | 1.06 (0.73-1.54) | 0.762 |  |
| Yes | ref | 0.77 (0.34-1.73) | 0.523 | 1.18 (0.59-2.37) | 0.641 |  |
| Tumor |  |  |  |  |  | 0.382 |
| No | ref | 0.86 (0.59-1.26) | 0.446 | 1.21 (0.86-1.72) | 0.270 |  |
| Yes | ref | 0.65 (0.20-2.15) | 0.486 | 0.54 (0.18-1.64) | 0.279 |  |
| EPTB |  |  |  |  |  | 0.906 |
| No | ref | 0.82 (0.51-1.32) | 0.422 | 1.27 (0.82-1.96) | 0.280 |  |
| Yes | ref | 0.89 (0.51-1.54) | 0.671 | 0.96 (0.59-1.58) | 0.886 |  |
| Cavity |  |  |  |  |  | 0.608 |
| No | ref | 0.85 (0.56-1.30) | 0.456 | 1.27 (0.87-1.86) | 0.214 |  |
| Yes | ref | 0.70 (0.36-1.36) | 0.295 | 0.68 (0.36-1.31) | 0.252 |  |

Note: PNI: Prognostic Nutritional Index; BMI: Body mass index; DST: Drug susceptibility testing; DS-TB: Drug-susceptible; MDR-TB: Multidrug-resistant TB; DR_nonMDR: Drug-resistant TB except MDR-TB; HBV: Hepatitis B virus; EPTB: Extrapulmonary tuberculosis.

Supplementary Table 15 Subgroup analysis of the association between SII and unfavorable treatment outcome

| Subgroup | HR (95%*CI*) | | | | | *P* for  interaction |
| --- | --- | --- | --- | --- | --- | --- |
|  | Q1 | Q2 | *P* | Q3 | *P* |  |
| Age group/years |  |  |  |  |  | 0.126 |
| <30 | ref | 1.59 (0.66-3.86) | 0.303 | 0.91 (0.32-2.58) | 0.862 |  |
| ≥30&<60 | ref | 1.22 (0.69-2.17) | 0.498 | 1.46 (0.84-2.54) | 0.177 |  |
| ≥60 | ref | 0.96 (0.53-1.76) | 0.900 | 1.87 (1.09-3.21) | 0.024 |  |
| Sex |  |  |  |  |  | 0.049 |
| Female | ref | 0.83 (0.45-1.52) | 0.540 | 0.94 (0.50-1.75) | 0.838 |  |
| Male | ref | 1.46 (0.90-2.38) | 0.128 | 2.10 (1.33-3.31) | 0.002 |  |
| BMI group/kg·m^-2^ |  |  |  |  |  | 0.157 |
| <18.5 | ref | 1.75 (0.72-4.22) | 0.215 | 3.09 (1.36-7.02) | 0.007 |  |
| ≥18.5&<24.0 | ref | 0.90 (0.55-1.47) | 0.666 | 1.21 (0.75-1.96) | 0.428 |  |
| ≥24.0 | ref | 1.59 (0.66-3.83) | 0.301 | 1.45 (0.58-3.59) | 0.425 |  |
| Smoking |  |  |  |  |  | 0.152 |
| No | ref | 0.86 (0.54-1.35) | 0.505 | 1.38 (0.91-2.11) | 0.131 |  |
| Yes | ref | 2.15 (1.07-4.34) | 0.032 | 2.26 (1.14-4.47) | 0.020 |  |
| Treatment Category |  |  |  |  |  | 0.664 |
| Initial | ref | 1.07 (0.70-1.64) | 0.751 | 1.50 (1.01-2.23) | 0.042 |  |
| Retreat | ref | 1.64 (0.71-3.78) | 0.244 | 2.20 (0.98-4.93) | 0.055 |  |
| DST |  |  |  |  |  | 0.182 |
| DS-TB | ref | 0.99 (0.65-1.50) | 0.952 | 1.33 (0.91-1.97) | 0.144 |  |
| MDR-TB | ref | 1.59 (0.54-4.66) | 0.398 | 1.61 (0.50-5.20) | 0.426 |  |
| DR_nonMDR | ref | 1.26 (0.25-6.35) | 0.778 | 4.53 (1.00-20.57) | 0.051 |  |
| Diabetes |  |  |  |  |  | 0.929 |
| No | ref | 1.12 (0.72-1.76) | 0.611 | 1.60 (1.06-2.43) | 0.026 |  |
| Yes | ref | 1.05 (0.52-2.13) | 0.885 | 1.36 (0.69-2.65) | 0.373 |  |
| HBV |  |  |  |  |  | 0.504 |
| No | ref | 1.11 (0.75-1.66) | 0.600 | 1.60 (1.11-2.32) | 0.012 |  |
| Yes | ref | 2.44 (0.63-9.43) | 0.197 | 1.55 (0.37-6.44) | 0.550 |  |
| Hypertension |  |  |  |  |  | 0.753 |
| No | ref | 1.06 (0.69-1.62) | 0.794 | 1.47 (0.99-2.18) | 0.056 |  |
| Yes | ref | 1.35 (0.61-3.01) | 0.457 | 1.84 (0.82-4.12) | 0.136 |  |
| Anemia |  |  |  |  |  | 0.471 |
| No | ref | 1.07 (0.70-1.62) | 0.755 | 1.54 (1.03-2.30) | 0.033 |  |
| Yes | ref | 1.41 (0.56-3.59) | 0.466 | 1.69 (0.73-3.89) | 0.218 |  |
| Tumor |  |  |  |  |  | 0.893 |
| No | ref | 1.15 (0.77-1.71) | 0.501 | 1.64 (1.13-2.39) | 0.010 |  |
| Yes | ref | 0.95 (0.30-3.06) | 0.935 | 1.11 (0.39-3.19) | 0.850 |  |
| EPTB |  |  |  |  |  | 0.770 |
| No | ref | 1.33 (0.81-2.18) | 0.268 | 1.84 (1.14-2.96) | 0.012 |  |
| Yes | ref | 0.84 (0.48-1.49) | 0.554 | 1.18 (0.69-2.03) | 0.537 |  |
| Cavity |  |  |  |  |  | 0.499 |
| No | ref | 1.23 (0.81-1.87) | 0.336 | 1.71 (0.81-2.10) | 0.158 |  |
| Yes | ref | 1.00 (0.43-2.36) | 0.996 | 1.53 (1.02-2.90) | 0.040 |  |

Note: SII: Systemic Immune-Inflammation Index; BMI: Body mass index; DST: Drug susceptibility testing; DS-TB: Drug-susceptible; MDR-TB: Multidrug-resistant TB; DR_nonMDR: Drug-resistant TB except MDR-TB; HBV: Hepatitis B virus; EPTB: Extrapulmonary tuberculosis.

Supplementary Table 16 Subgroup analysis of the association between SIRI and unfavorable treatment outcome

| Subgroup | HR (95%*CI*) | | | | | *P* for  interaction |
| --- | --- | --- | --- | --- | --- | --- |
|  | Q1 | Q2 | *P* | Q3 | *P* |  |
| Age group/years |  |  |  |  |  | 0.022 |
| <30 | ref | 1.31 (0.56-3.05) | 0.538 | 0.59 (0.21-1.69) | 0.324 |  |
| ≥30&<60 | ref | 1.18 (0.65-2.13) | 0.589 | 1.74 (0.97-3.09) | 0.061 |  |
| ≥60 | ref | 1.87 (0.84-4.15) | 0.126 | 3.31 (1.56-7.05) | 0.002 |  |
| Sex |  |  |  |  |  | 0.490 |
| Female | ref | 1.31 (0.70-2.44) | 0.398 | 1.47 (0.78-2.76) | 0.234 |  |
| Male | ref | 1.29 (0.75-2.21) | 0.354 | 2.00 (1.20-3.30) | 0.007 |  |
| BMI group/kg·m^-2^ |  |  |  |  |  | 0.101 |
| <18.5 | ref | 2.29 (0.90-5.83) | 0.081 | 3.74 (1.55-9.05) | 0.003 |  |
| ≥18.5&<24.0 | ref | 1.30 (0.74-2.26) | 0.358 | 1.66 (0.96-2.86) | 0.068 |  |
| ≥24.0 | ref | 0.73 (0.30-1.75) | 0.478 | 1.31 (0.56-3.06) | 0.532 |  |
| Smoking |  |  |  |  |  | 0.215 |
| No | ref | 1.47 (0.91-2.39) | 0.114 | 1.92 (1.21-3.06) | 0.006 |  |
| Yes | ref | 0.84 (0.40-1.74) | 0.632 | 1.44 (0.74-2.81) | 0.285 |  |
| Treatment Category |  |  |  |  |  | 0.633 |
| Initial | ref | 1.10 (0.70-1.71) | 0.679 | 1.64 (1.08-2.49) | 0.021 |  |
| Retreat | ref | 1.65 (0.61-4.43) | 0.323 | 2.66 (1.04-6.81) | 0.041 |  |
| DST |  |  |  |  |  | 0.124 |
| DS-TB | ref | 0.95 (0.31-2.88) | 0.927 | 1.15 (0.73-1.81) | 0.555 |  |
| MDR-TB | ref | 1.36 (0.46-4.03) | 0.582 | 1.72 (1.12-5.65) | 0.013 |  |
| DR_nonMDR | ref | 7.44 (0.85-65.40) | 0.070 | 8.68 (0.93-81.18) | 0.058 |  |
| Diabetes |  |  |  |  |  | 0.500 |
| No | ref | 1.24 (0.77-2.00) | 0.377 | 1.97 (1.26-3.10) | 0.003 |  |
| Yes | ref | 1.10 (0.52-2.32) | 0.809 | 1.28 (0.62-2.62) | 0.501 |  |
| HBV |  |  |  |  |  | 0.220 |
| No | ref | 1.29 (0.84-1.99) | 0.243 | 1.96 (1.30-2.95) | 0.001 |  |
| Yes | ref | 0.91 (0.26-3.20) | 0.879 | 0.78 (0.19-3.25) | 0.738 |  |
| Hypertension |  |  |  |  |  | 0.469 |
| No | ref | 1.31 (0.84-2.04) | 0.227 | 1.65 (1.08-2.53) | 0.022 |  |
| Yes | ref | 0.96 (0.37-2.49) | 0.931 | 2.14 (0.87-5.29) | 0.099 |  |
| Anemia |  |  |  |  |  | 0.059 |
| No | ref | 1.26 (0.82-1.92) | 0.294 | 1.53 (1.01-2.32) | 0.047 |  |
| Yes | ref | 1.42 (0.38-5.29) | 0.603 | 3.29 (0.98-11.11) | 0.055 |  |
| Tumor |  |  |  |  |  | 0.547 |
| No | ref | 1.15 (0.75-1.75) | 0.520 | 1.72 (1.15-2.56) | 0.008 |  |
| Yes | ref | 3.08 (0.59-16.11) | 0.182 | 3.53 (0.72-17.40) | 0.121 |  |
| EPTB |  |  |  |  |  | 0.976 |
| No | ref | 1.25 (0.73-2.16) | 0.418 | 1.97 (1.18-3.28) | 0.009 |  |
| Yes | ref | 1.24 (0.67-2.26) | 0.493 | 1.58 (0.87-2.84) | 0.132 |  |
| Cavity |  |  |  |  |  | 0.846 |
| No | ref | 1.27 (0.81-2.01) | 0.300 | 1.45 (0.93-2.82) | 0.383 |  |
| Yes | ref | 1.16 (0.48-2.81) | 0.744 | 1.95 (1.26-3.01) | 0.003 |  |

Note: SIRI: Systemic Inflammatory Response Index; BMI: Body mass index; DST: Drug susceptibility testing; DS-TB: Drug-susceptible; MDR-TB: Multidrug-resistant TB; DR_nonMDR: Drug-resistant TB except MDR-TB; HBV: Hepatitis B virus; EPTB: Extrapulmonary tuberculosis.

Supplementary Table 17 Cox regression analysis between inflammatory status and unfavorable treatment outcomes among patients without comorbidities

| Inflammatory  indices | Model 1 | | Model 2 | | Model 3 | |
| --- | --- | --- | --- | --- | --- | --- |
|  | HR (95% *CI*) | *P* | HR (95% *CI*) | *P* | HR (95% *CI*) | *P* |
| CAR |  |  |  |  |  |  |
| Continuous* | 1.36 (1.19-1.54) | <0.001 | 1.27 (1.11-1.45) | <0.001 | 1.28 (1.12-1.47) | <0.001 |
| Q1 | ref |  | ref |  | ref |  |
| Q2 | 1.00 (0.55-1.81) | 0.990 | 0.91 (0.50-1.66) | 0.748 | 1.00 (0.54-1.85) | 0.991 |
| Q3 | 2.30 (1.38-3.84) | 0.001 | 1.79 (1.05-3.05) | 0.034 | 1.97 (1.13-3.44) | 0.017 |
| *P* for trend |  | <0.001 |  | 0.024 |  | 0.012 |
| CLR |  |  |  |  |  |  |
| Continuous* | 1.15 (1.09-1.22) | <0.001 | 1.09 (1.02-1.16) | 0.008 | 1.12 (1.04-1.19) | 0.001 |
| Q1 | ref |  | ref |  | ref |  |
| Q2 | 1.04 (0.58-1.88) | 0.898 | 0.93 (0.51-1.68) | 0.805 | 1.03 (0.56-1.89) | 0.918 |
| Q3 | 2.19 (1.31-3.65) | 0.003 | 1.65 (0.96-2.83) | 0.069 | 1.82 (1.04-3.19) | 0.035 |
| *P* for trend |  | 0.002 |  | 0.054 |  | 0.027 |
| dNLR |  |  |  |  |  |  |
| Continuous* | 1.27 (1.16-1.40) | <0.001 | 1.16 (1.05-1.28) | 0.003 | 1.20 (1.08-1.33) | <0.001 |
| Q1 | ref |  | ref |  | ref |  |
| Q2 | 0.87 (0.47-1.58) | 0.638 | 0.79 (0.43-1.44) | 0.437 | 0.82 (0.45-1.51) | 0.532 |
| Q3 | 2.07 (1.25-3.43) | 0.005 | 1.60 (0.95-2.68) | 0.077 | 1.69 (1.00-2.86) | 0.050 |
| *P* for trend |  | 0.003 |  | 0.055 |  | 0.039 |
| ELR |  |  |  |  |  |  |
| Continuous* | 1.17 (1.02-1.35) | 0.026 | 1.07 (0.91-1.26) | 0.407 | 1.03 (0.88-1.22) | 0.696 |
| Q1 | ref |  | ref |  | ref |  |
| Q2 | 0.58 (0.33-1.02) | 0.060 | 0.56 (0.31-0.99) | 0.045 | 0.55 (0.31-0.97) | 0.040 |
| Q3 | 1.05 (0.65-1.71) | 0.841 | 0.81 (0.49-1.34) | 0.419 | 0.78 (0.47-1.30) | 0.342 |
| *P* for trend |  | 0.806 |  | 0.459 |  | 0.374 |
| EMR |  |  |  |  |  |  |
| Continuous* | 1.01 (0.81-1.24) | 0.963 | 1.01 (0.78-1.30) | 0.966 | 0.98 (0.65-1.47) | 0.913 |
| Q1 | ref |  | ref |  | ref |  |
| Q2 | 1.13 (0.67-1.91) | 0.650 | 1.20 (0.71-2.03) | 0.496 | 1.12 (0.66-1.90) | 0.673 |
| Q3 | 0.93 (0.55-1.59) | 0.803 | 0.97 (0.57-1.65) | 0.901 | 0.92 (0.54-1.58) | 0.763 |
| *P* for trend |  | 0.787 |  | 0.881 |  | 0.744 |
| ENR |  |  |  |  |  |  |
| Continuous* | 0.99 (0.80-1.23) | 0.950 | 0.97 (0.79-1.20) | 0.795 | 0.94 (0.77-1.14) | 0.543 |
| Q1 | ref |  | ref |  | ref |  |
| Q2 | 0.67 (0.39-1.15) | 0.142 | 0.69 (0.40-1.19) | 0.179 | 0.61 (0.35-1.07) | 0.086 |
| Q3 | 0.78 (0.47-1.28) | 0.327 | 0.79 (0.48-1.30) | 0.348 | 0.75 (0.45-1.24) | 0.262 |
| *P* for trend |  | 0.337 |  | 0.359 |  | 0.283 |
| MLR |  |  |  |  |  |  |
| Continuous* | 1.24 (1.07-1.42) | 0.003 | 1.08 (0.92-1.26) | 0.355 | 1.09 (0.93-1.28) | 0.282 |
| Q1 | ref |  | ref |  | ref |  |
| Q2 | 0.92 (0.52-1.62) | 0.772 | 0.82 (0.46-1.45) | 0.493 | 0.84 (0.47-1.49) | 0.557 |
| Q3 | 1.63 (0.98-2.70) | 0.058 | 1.14 (0.67-1.95) | 0.626 | 1.18 (0.69-2.05) | 0.544 |
| *P* for trend |  | 0.053 |  | 0.588 |  | 0.519 |
| NLR |  |  |  |  |  |  |
| Continuous* | 1.14 (1.06-1.22) | <0.001 | 1.06 (0.98-1.14) | 0.127 | 1.08 (1.00-1.17) | 0.059 |
| Q1 | ref |  | ref |  | ref |  |
| Q2 | 0.64 (0.34-1.22) | 0.174 | 0.56 (0.30-1.07) | 0.080 | 0.60 (0.31-1.14) | 0.119 |
| Q3 | 2.21 (1.35-3.62) | 0.002 | 1.63 (0.97-2.72) | 0.063 | 1.73 (1.02-2.91) | 0.041 |
| *P* for trend |  | <0.001 |  | 0.035 |  | 0.027 |
| PLR |  |  |  |  |  |  |
| Continuous* | 1.13 (1.01-1.27) | 0.031 | 1.03 (0.91-1.17) | 0.604 | 1.06 (0.94-1.19) | 0.323 |
| Q1 | ref |  | ref |  | ref |  |
| Q2 | 0.83 (0.47-1.44) | 0.504 | 0.79 (0.45-1.38) | 0.413 | 0.95 (0.53-1.68) | 0.854 |
| Q3 | 1.24 (0.75-2.07) | 0.397 | 0.95 (0.56-1.60) | 0.842 | 1.16 (0.67-1.99) | 0.595 |
| *P* for trend |  | 0.372 |  | 0.877 |  | 0.577 |
| PNI |  |  |  |  |  |  |
| Continuous* | 1.16 (1.07-1.26) | <0.001 | 1.07 (0.98-1.18) | 0.124 | 1.10 (1.01-1.20) | 0.037 |
| Q1 | ref |  | ref |  | ref |  |
| Q2 | 0.90 (0.52-1.57) | 0.713 | 0.85 (0.49-1.48) | 0.559 | 0.92 (0.52-1.60) | 0.761 |
| Q3 | 1.31 (0.78-2.20) | 0.300 | 0.99 (0.58-1.68) | 0.970 | 1.17 (0.68-2.01) | 0.559 |
| *P* for trend |  | 0.275 |  | 0.995 |  | 0.536 |
| SII |  |  |  |  |  |  |
| Continuous* | 1.19 (1.09-1.31) | <0.001 | 1.10 (0.99-1.21) | 0.073 | 1.12 (1.01-1.24) | 0.034 |
| Q1 | ref |  | ref |  | ref |  |
| Q2 | 0.86 (0.48-1.53) | 0.607 | 0.79 (0.44-1.41) | 0.420 | 0.89 (0.49-1.60) | 0.693 |
| Q3 | 1.67 (1.01-2.77) | 0.047 | 1.38 (0.82-2.32) | 0.226 | 1.52 (0.89-2.59) | 0.129 |
| *P* for trend |  | 0.038 |  | 0.184 |  | 0.112 |
| SIRI |  |  |  |  |  |  |
| Continuous* | 1.17 (1.08-1.28) | <0.001 | 1.09 (1.00-1.20) | 0.059 | 1.12 (1.02-1.24) | 0.024 |
| Q1 | ref |  | ref |  | ref |  |
| Q2 | 1.26 (0.72-2.22) | 0.414 | 1.10 (0.63-1.94) | 0.738 | 1.10 (0.62-1.94) | 0.746 |
| Q3 | 1.88 (1.11-3.19) | 0.019 | 1.43 (0.83-2.48) | 0.198 | 1.43 (0.82-2.51) | 0.211 |
| *P* for trend |  | 0.017 |  | 0.187 |  | 0.202 |

Note: *: for each one standard deviation (SD) increase. Model 1: Non-adjusted model; Model 1 adjusted for: Age, Sex, and BMI; Model 3 adjusted for: Age, Sex, BMI, Work Status, Drinking, Smoking, Treatment Category, Cavity, and DST. BMI: Body Mass Index; DST: Drug susceptibility testing; CAR: C-reactive protein to albumin ratio; CLR: C-reactive protein to lymphocyte ratio; dNLR: Derived neutrophil to lymphocyte ratio; ELR: Eosinophil to lymphocyte ratio; EMR: Eosinophil to monocyte ratio; ENR: Eosinophil to neutrophil ratio; MLR: Monocyte to lymphocyte ratio; NLR: Neutrophil to lymphocyte ratio; PLR: Platelet to lymphocyte ratio; PNI: Prognostic Nutritional Index; SII: Systemic Immune-Inflammation Index; SIRI: Systemic Inflammatory Response Index.

Supplementary Table 18 Cox regression analysis between inflammatory status and unfavorable treatment outcomes among patients with complete data

| Inflammatory  indices | Model 1 | | Model 2 | | Model 3 | |
| --- | --- | --- | --- | --- | --- | --- |
|  | HR (95% *CI*) | *P* | HR (95% *CI*) | *P* | HR (95% *CI*) | *P* |
| CAR |  |  |  |  |  |  |
| Continuous* | 1.35 (1.23-1.48) | <0.001 | 1.24 (1.12-1.38) | <0.001 | 1.25 (1.12-1.39) | <0.001 |
| Q1 | ref |  | ref |  | ref |  |
| Q2 | 1.40 (0.89-2.19) | 0.148 | 1.08 (0.69-1.71) | 0.737 | 1.08 (0.68-1.72) | 0.749 |
| Q3 | 2.51 (1.67-3.79) | <0.001 | 1.57 (1.02-2.42) | 0.039 | 1.52 (0.97-2.38) | 0.069 |
| *P* for trend |  | <0.001 |  | 0.023 |  | 0.041 |
| CLR |  |  |  |  |  |  |
| Continuous* | 1.18 (1.11-1.26) | <0.001 | 1.08 (1.01-1.16) | 0.027 | 1.10 (1.01-1.18) | 0.02 |
| Q1 | ref |  | ref |  | ref |  |
| Q2 | 1.80 (1.13-2.84) | 0.013 | 1.35 (0.85-2.16) | 0.202 | 1.34 (0.83-2.16) | 0.227 |
| Q3 | 2.84 (1.84-4.36) | <0.001 | 1.74 (1.10-2.73) | 0.017 | 1.67 (1.04-2.67) | 0.033 |
| *P* for trend |  | <0.001 |  | 0.014 |  | 0.03 |
| dNLR |  |  |  |  |  |  |
| Continuous* | 1.31 (1.22-1.40) | <0.001 | 1.18 (1.10-1.27) | <0.001 | 1.21 (1.11-1.31) | <0.001 |
| Q1 | ref |  | ref |  | ref |  |
| Q2 | 1.59 (0.97-2.59) | 0.064 | 1.29 (0.79-2.10) | 0.316 | 1.30 (0.79-2.14) | 0.294 |
| Q3 | 3.33 (2.15-5.15) | <0.001 | 2.36 (1.51-3.69) | <0.001 | 2.27 (1.44-3.57) | <0.001 |
| *P* for trend |  | <0.001 |  | <0.001 |  | <0.001 |
| ELR |  |  |  |  |  |  |
| Continuous* | 1.10 (1.01-1.20) | 0.026 | 1.00 (0.89-1.12) | 0.977 | 0.97 (0.86-1.10) | 0.672 |
| Q1 | ref |  | ref |  | ref |  |
| Q2 | 0.84 (0.56-1.25) | 0.385 | 0.83 (0.56-1.24) | 0.375 | 0.92 (0.62-1.38) | 0.7 |
| Q3 | 1.20 (0.83-1.73) | 0.337 | 0.90 (0.62-1.31) | 0.585 | 0.93 (0.64-1.36) | 0.705 |
| *P* for trend |  | 0.309 |  | 0.614 |  | 0.711 |
| EMR |  |  |  |  |  |  |
| Continuous* | 0.88 (0.73-1.05) | 0.158 | 0.87 (0.74-1.03) | 0.115 | 0.87 (0.74-1.03) | 0.105 |
| Q1 | ref |  | ref |  | ref |  |
| Q2 | 0.78 (0.55-1.12) | 0.177 | 0.84 (0.58-1.20) | 0.333 | 0.91 (0.63-1.31) | 0.615 |
| Q3 | 0.58 (0.39-0.86) | 0.007 | 0.62 (0.42-0.93) | 0.019 | 0.64 (0.43-0.96) | 0.032 |
| *P* for trend |  | 0.007 |  | 0.019 |  | 0.035 |
| ENR |  |  |  |  |  |  |
| Continuous* | 0.79 (0.63-0.98) | 0.034 | 0.79 (0.64-0.98) | 0.029 | 0.80 (0.66-0.98) | 0.032 |
| Q1 | ref |  | ref |  | ref |  |
| Q2 | 0.72 (0.50-1.03) | 0.072 | 0.74 (0.51-1.06) | 0.101 | 0.77 (0.53-1.11) | 0.16 |
| Q3 | 0.56 (0.38-0.82) | 0.003 | 0.59 (0.40-0.86) | 0.007 | 0.61 (0.41-0.90) | 0.014 |
| *P* for trend |  | 0.003 |  | 0.006 |  | 0.012 |
| MLR |  |  |  |  |  |  |
| Continuous* | 1.39 (1.28-1.52) | <0.001 | 1.23 (1.12-1.36) | <0.001 | 1.22 (1.10-1.36) | <0.001 |
| Q1 | ref |  | ref |  | ref |  |
| Q2 | 1.02 (0.63-1.64) | 0.938 | 0.81 (0.50-1.30) | 0.375 | 0.77 (0.48-1.25) | 0.295 |
| Q3 | 2.53 (1.69-3.78) | <0.001 | 1.60 (1.04-2.44) | 0.032 | 1.40 (0.91-2.16) | 0.126 |
| *P* for trend |  | <0.001 |  | 0.006 |  | 0.038 |
| NLR |  |  |  |  |  |  |
| Continuous* | 1.16 (1.11-1.21) | <0.001 | 1.09 (1.03-1.14) | <0.001 | 1.11 (1.05-1.17) | <0.001 |
| Q1 | ref |  | ref |  | ref |  |
| Q2 | 1.35 (0.82-2.22) | 0.234 | 1.09 (0.66-1.79) | 0.739 | 1.15 (0.70-1.91) | 0.583 |
| Q3 | 3.31 (2.16-5.07) | <0.001 | 2.15 (1.38-3.35) | <0.001 | 2.03 (1.29-3.18) | 0.002 |
| *P* for trend |  | <0.001 |  | <0.001 |  | <0.001 |
| PLR |  |  |  |  |  |  |
| Continuous* | 1.13 (1.03-1.24) | 0.008 | 1.02 (0.92-1.14) | 0.672 | 1.03 (0.92-1.16) | 0.589 |
| Q1 | ref |  | ref |  | ref |  |
| Q2 | 1.26 (0.85-1.86) | 0.253 | 1.14 (0.77-1.69) | 0.521 | 1.20 (0.81-1.78) | 0.372 |
| Q3 | 1.29 (0.87-1.91) | 0.204 | 0.92 (0.61-1.39) | 0.705 | 0.92 (0.61-1.40) | 0.706 |
| *P* for trend |  | 0.212 |  | 0.653 |  | 0.66 |
| PNI |  |  |  |  |  |  |
| Continuous* | 1.16 (1.08-1.24) | <0.001 | 1.06 (0.98-1.15) | 0.153 | 1.07 (0.98-1.17) | 0.108 |
| Q1 | ref |  | ref |  | ref |  |
| Q2 | 0.87 (0.58-1.30) | 0.491 | 0.82 (0.55-1.24) | 0.355 | 0.88 (0.58-1.33) | 0.552 |
| Q3 | 1.39 (0.96-2.01) | 0.084 | 1.05 (0.72-1.53) | 0.816 | 1.08 (0.74-1.59) | 0.68 |
| *P* for trend |  | 0.067 |  | 0.745 |  | 0.639 |
| SII |  |  |  |  |  |  |
| Continuous* | 1.22 (1.13-1.32) | <0.001 | 1.12 (1.04-1.22) | 0.004 | 1.14 (1.05-1.25) | 0.003 |
| Q1 | ref |  | ref |  | ref |  |
| Q2 | 1.37 (0.88-2.13) | 0.166 | 1.09 (0.70-1.70) | 0.711 | 1.18 (0.75-1.85) | 0.467 |
| Q3 | 2.23 (1.49-3.33) | <0.001 | 1.65 (1.09-2.50) | 0.017 | 1.61 (1.06-2.45) | 0.025 |
| *P* for trend |  | <0.001 |  | 0.009 |  | 0.019 |
| SIRI |  |  |  |  |  |  |
| Continuous* | 1.25 (1.18-1.32) | <0.001 | 1.24 (1.16-1.32) | <0.001 | 1.23 (1.15-1.33) | <0.001 |
| Q1 | ref |  | ref |  | ref |  |
| Q2 | 1.64 (1.01-2.66) | 0.046 | 1.25 (0.77-2.04) | 0.37 | 1.18 (0.72-1.93) | 0.523 |
| Q3 | 3.06 (1.98-4.73) | <0.001 | 2.04 (1.30-3.22) | 0.002 | 1.76 (1.11-2.81) | 0.017 |
| *P* for trend |  | <0.001 |  | <0.001 |  | 0.007 |

Note: *: for each one standard deviation (SD) increase. Model 1: Non-adjusted model; Model 1 adjusted for: Age, Sex, and BMI; Model 3 adjusted for: Age, Sex, BMI, Work Status, Smoking, Treatment Category, Fatigue, Weight Loss, Diabetes, Hypertension, Tumor, Anemia, Cavity, DST, and MTB2. BMI: Body Mass Index; DST: Drug susceptibility testing; MTB2: Bacteriological test result at 2-month treatment; CAR: C-reactive protein to albumin ratio; CLR: C-reactive protein to lymphocyte ratio; dNLR: Derived neutrophil to lymphocyte ratio; ELR: Eosinophil to lymphocyte ratio; EMR: Eosinophil to monocyte ratio; ENR: Eosinophil to neutrophil ratio; MLR: Monocyte to lymphocyte ratio; NLR: Neutrophil to lymphocyte ratio; PLR: Platelet to lymphocyte ratio; PNI: Prognostic Nutritional Index; SII: Systemic Immune-Inflammation Index; SIRI: Systemic Inflammatory Response Index.

Supplementary Table 19 Area under the curve for ROC analysis among patients without comorbidities

| Model | 6-month AUC | *P* | 9-month AUC | *P* | 12-month AUC | *P* |
| --- | --- | --- | --- | --- | --- | --- |
| Basic model | 0.751 | ref | 0.744 | ref | 0.718 | ref |
| +CAR | 0.775 | 0.277 | 0.759 | 0.852 | 0.732 | 0.860 |
| +CLR | 0.771 | 0.776 | 0.762 | 0.829 | 0.738 | 0.603 |
| +dNLR | 0.798 | 0.107 | 0.775 | 0.188 | 0.731 | 0.869 |
| +ELR | 0.783 | 0.163 | 0.764 | 0.766 | 0.738 | 0.587 |
| +EMR | 0.781 | 0.214 | 0.768 | 0.260 | 0.739 | 0.388 |
| +ENR | 0.798 | 0.078 | 0.776 | 0.154 | 0.740 | 0.371 |
| +MLR | 0.755 | 0.994 | 0.757 | 0.869 | 0.741 | 0.332 |
| +NLR | 0.800 | 0.058 | 0.779 | 0.147 | 0.738 | 0.538 |
| +PLR | 0.758 | 0.898 | 0.755 | 0.883 | 0.730 | 0.875 |
| +PNI | 0.770 | 0.801 | 0.764 | 0.650 | 0.746 | 0.222 |
| +SII | 0.794 | 0.114 | 0.780 | 0.122 | 0.731 | 0.869 |
| +SIRI | 0.763 | 0.875 | 0.751 | 0.898 | 0.733 | 0.851 |
| Combined model | 0.852 | 0.001 | 0.830 | 0.001 | 0.782 | 0.032 |

Note: Basic model: included age, sex, BMI, work status, drinking, smoking, treatment category, cavity, and DST. Combined model: a combination of blood inflammatory indices, added to the characteristics included in the basic model. BMI: Body Mass Index; DST: Drug susceptibility testing; AUC: Area under curve; CAR: C-reactive protein to albumin ratio; CLR: C-reactive protein to lymphocyte ratio; dNLR: Derived neutrophil to lymphocyte ratio; ELR: Eosinophil to lymphocyte ratio; EMR: Eosinophil to monocyte ratio; ENR: Eosinophil to neutrophil ratio; MLR: Monocyte to lymphocyte ratio; NLR: Neutrophil to lymphocyte ratio; PLR: Platelet to lymphocyte ratio; PNI: Prognostic Nutritional Index; SII: Systemic Immune-Inflammation Index; SIRI: Systemic Inflammatory Response Index.

Supplementary Table 20 Area under the curve for ROC analysis among patients with complete data

| Model | 6-month AUC | *P* | 9-month AUC | *P* | 12-month AUC | *P* |
| --- | --- | --- | --- | --- | --- | --- |
| Basic model | 0.765 | ref | 0.763 | ref | 0.722 | ref |
| +CAR | 0.770 | 0.894 | 0.783 | 0.675 | 0.753 | 0.230 |
| +CLR | 0.773 | 0.874 | 0.784 | 0.647 | 0.751 | 0.268 |
| +dNLR | 0.785 | 0.704 | 0.777 | 0.733 | 0.753 | 0.187 |
| +ELR | 0.765 | 1.000 | 0.773 | 0.859 | 0.744 | 0.534 |
| +EMR | 0.765 | 1.000 | 0.770 | 0.881 | 0.741 | 0.706 |
| +ENR | 0.768 | 0.916 | 0.769 | 0.887 | 0.739 | 0.728 |
| +MLR | 0.768 | 0.916 | 0.775 | 0.808 | 0.752 | 0.236 |
| +NLR | 0.786 | 0.654 | 0.782 | 0.726 | 0.757 | 0.035 |
| +PLR | 0.769 | 0.899 | 0.776 | 0.743 | 0.746 | 0.481 |
| +PNI | 0.777 | 0.826 | 0.777 | 0.728 | 0.747 | 0.435 |
| +SII | 0.765 | 1.000 | 0.773 | 0.859 | 0.745 | 0.495 |
| +SIRI | 0.775 | 0.860 | 0.774 | 0.853 | 0.749 | 0.341 |
| Combined model | 0.803 | 0.024 | 0.797 | 0.035 | 0.766 | 0.017 |

Note: Basic model: included age, sex, BMI, work status, smoking, treatment category, fatigue, weight loss, diabetes, hypertension, tumor, anemia, cavity, DST, and MTB2. Combined model: a combination of blood inflammatory indices, added to the characteristics included in the basic model. BMI: Body Mass Index; DST: Drug susceptibility testing; MTB2: Bacteriological test result at 2-month treatment; AUC: Area under curve; CAR: C-reactive protein to albumin ratio; CLR: C-reactive protein to lymphocyte ratio; dNLR: Derived neutrophil to lymphocyte ratio; ELR: Eosinophil to lymphocyte ratio; EMR: Eosinophil to monocyte ratio; ENR: Eosinophil to neutrophil ratio; MLR: Monocyte to lymphocyte ratio; NLR: Neutrophil to lymphocyte ratio; PLR: Platelet to lymphocyte ratio; PNI: Prognostic Nutritional Index; SII: Systemic Immune-Inflammation Index; SIRI: Systemic Inflammatory Response Index.


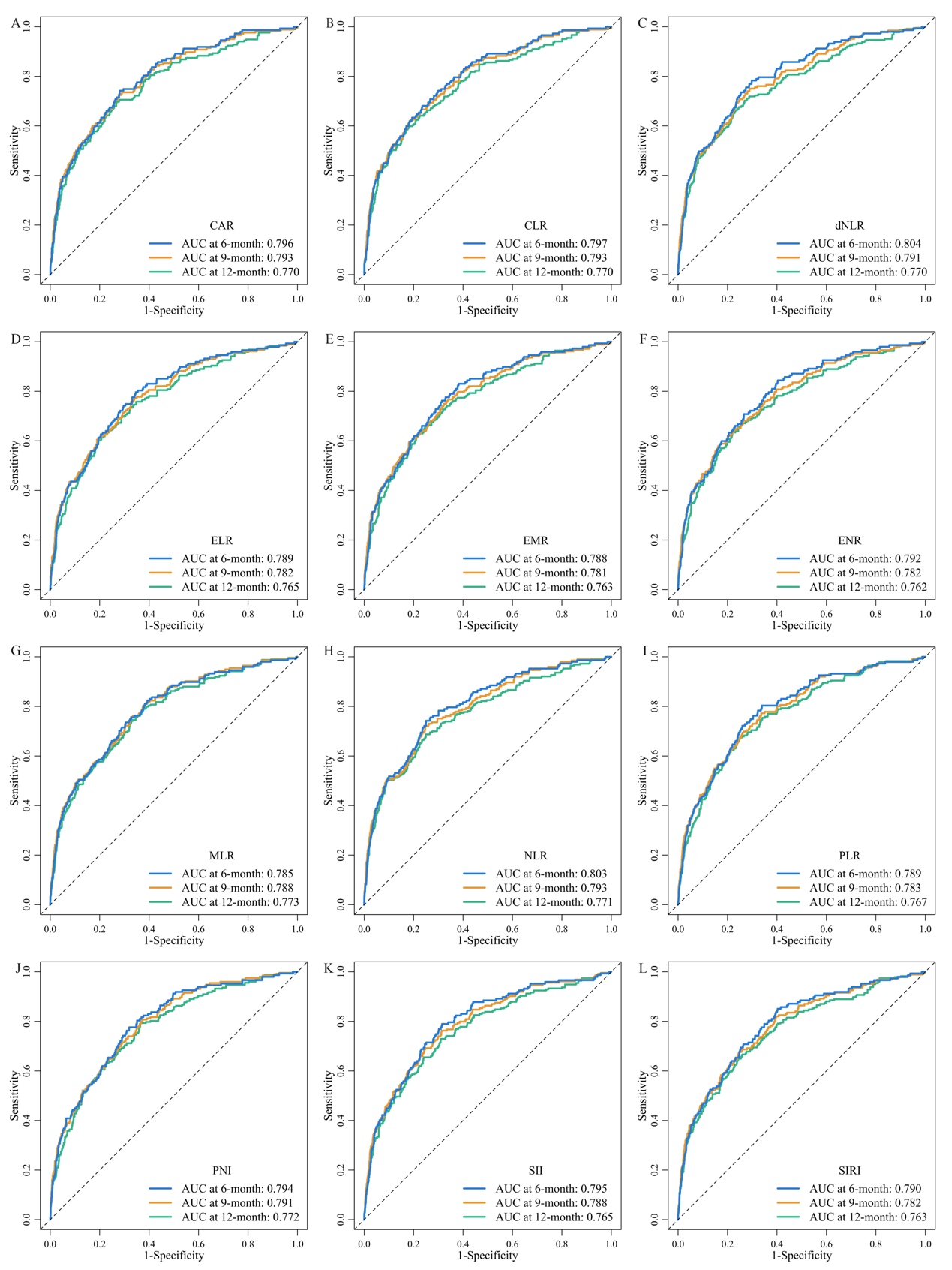


Supplementary Figure 1 Predictive value of single blood inflammatory status for treatment outcome. (A) CAR: C-reactive protein to albumin ratio; (B) CLR: C-reactive protein to lymphocyte ratio; (C) dNLR: Derived neutrophil to lymphocyte ratio; (D) ELR: Eosinophil to lymphocyte ratio; (E) EMR: Eosinophil to monocyte ratio; (F) ENR: Eosinophil to neutrophil ratio; (G) MLR: Monocyte to lymphocyte ratio; (H) NLR: Neutrophil to lymphocyte ratio; (I) PLR: Platelet to lymphocyte ratio; (J) PNI: Prognostic Nutritional Index; (K) SII: Systemic Immune-Inflammation Index; (L) SIRI: Systemic Inflammatory Response Index. AUC: Area under curve.


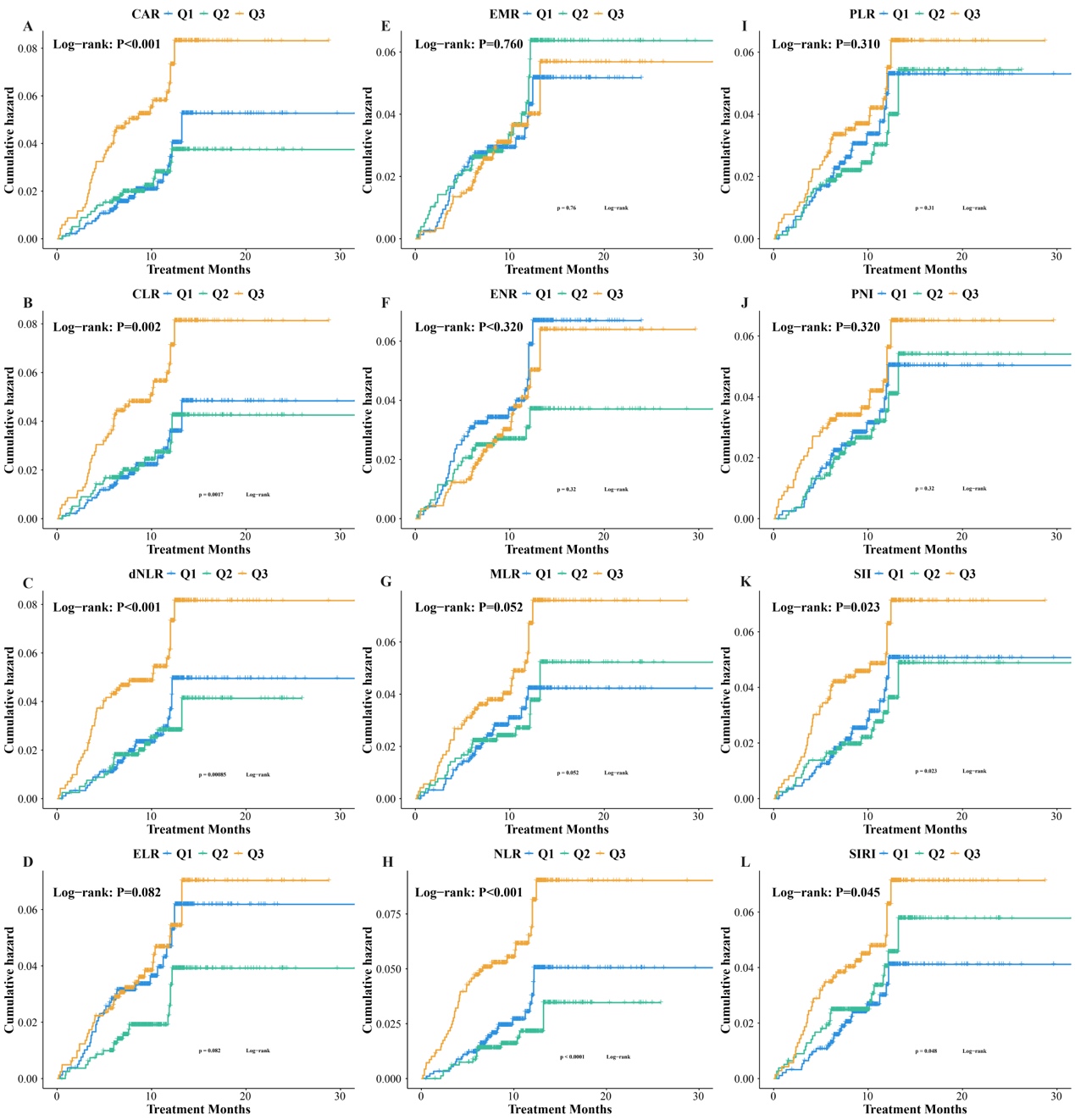


Supplementary Figure 2 Kaplan-Meier analysis among patients without comorbidities in different inflammatory status stratified by (A) CAR: C-reactive protein to albumin ratio; (B) CLR: C-reactive protein to lymphocyte ratio; (C) dNLR: Derived neutrophil to lymphocyte ratio; (D) ELR: Eosinophil to lymphocyte ratio; (E) EMR: Eosinophil to monocyte ratio; (F) ENR: Eosinophil to neutrophil ratio; (G) MLR: Monocyte to lymphocyte ratio; (H) NLR: Neutrophil to lymphocyte ratio; (I) PLR: Platelet to lymphocyte ratio; (J) PNI: Prognostic Nutritional Index; (K) SII: Systemic Immune-Inflammation Index; (L) SIRI: Systemic Inflammatory Response Index.


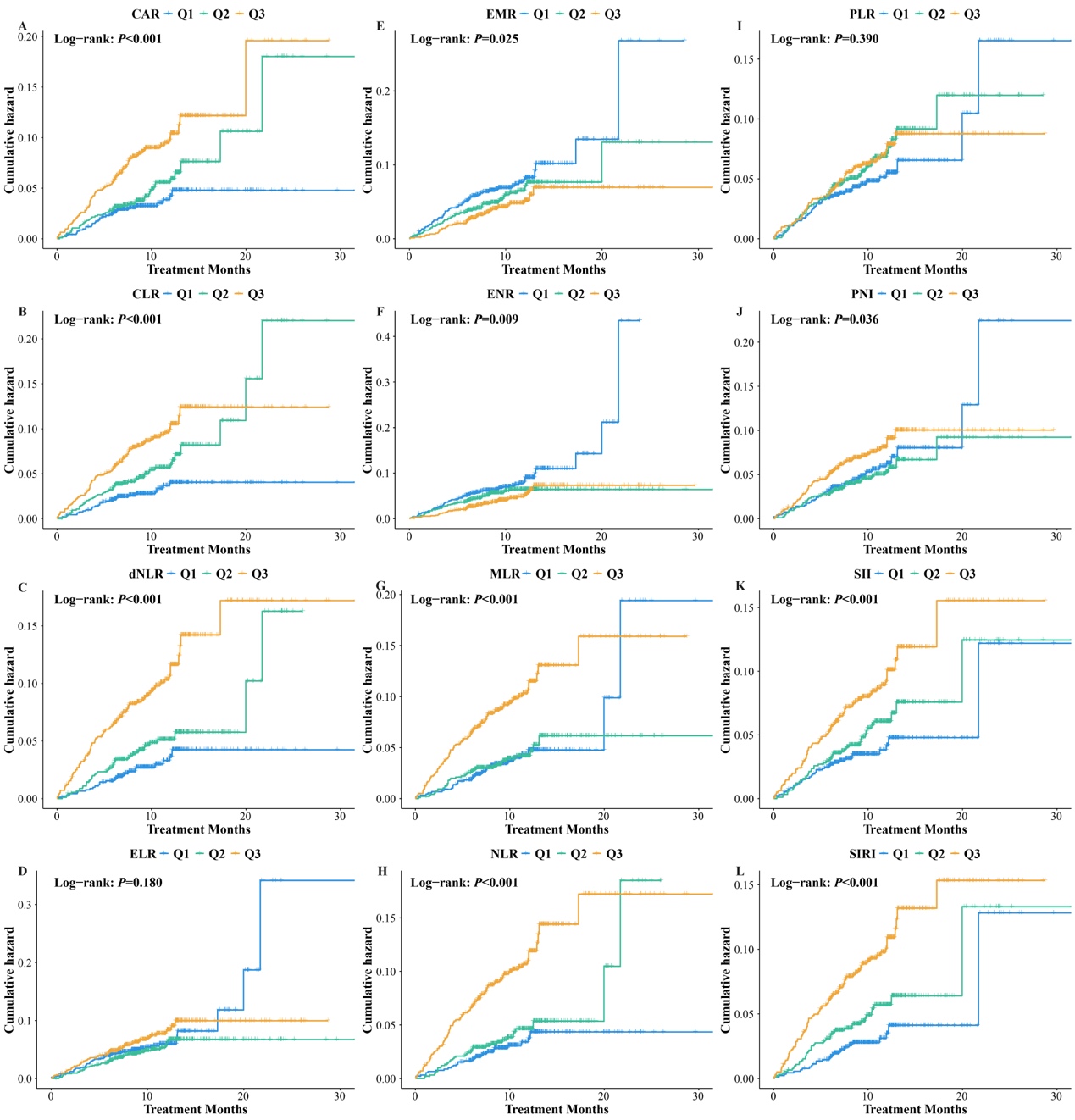


Supplementary Figure 3 Kaplan-Meier analysis among patients with complete data in different inflammatory status stratified by (A) CAR: C-reactive protein to albumin ratio; (B) CLR: C-reactive protein to lymphocyte ratio; (C) dNLR: Derived neutrophil to lymphocyte ratio; (D) ELR: Eosinophil to lymphocyte ratio; (E) EMR: Eosinophil to monocyte ratio; (F) ENR: Eosinophil to neutrophil ratio; (G) MLR: Monocyte to lymphocyte ratio; (H) NLR: Neutrophil to lymphocyte ratio; (I) PLR: Platelet to lymphocyte ratio; (J) PNI: Prognostic Nutritional Index; (K) SII: Systemic Immune-Inflammation Index; (L) SIRI: Systemic Inflammatory Response Index.


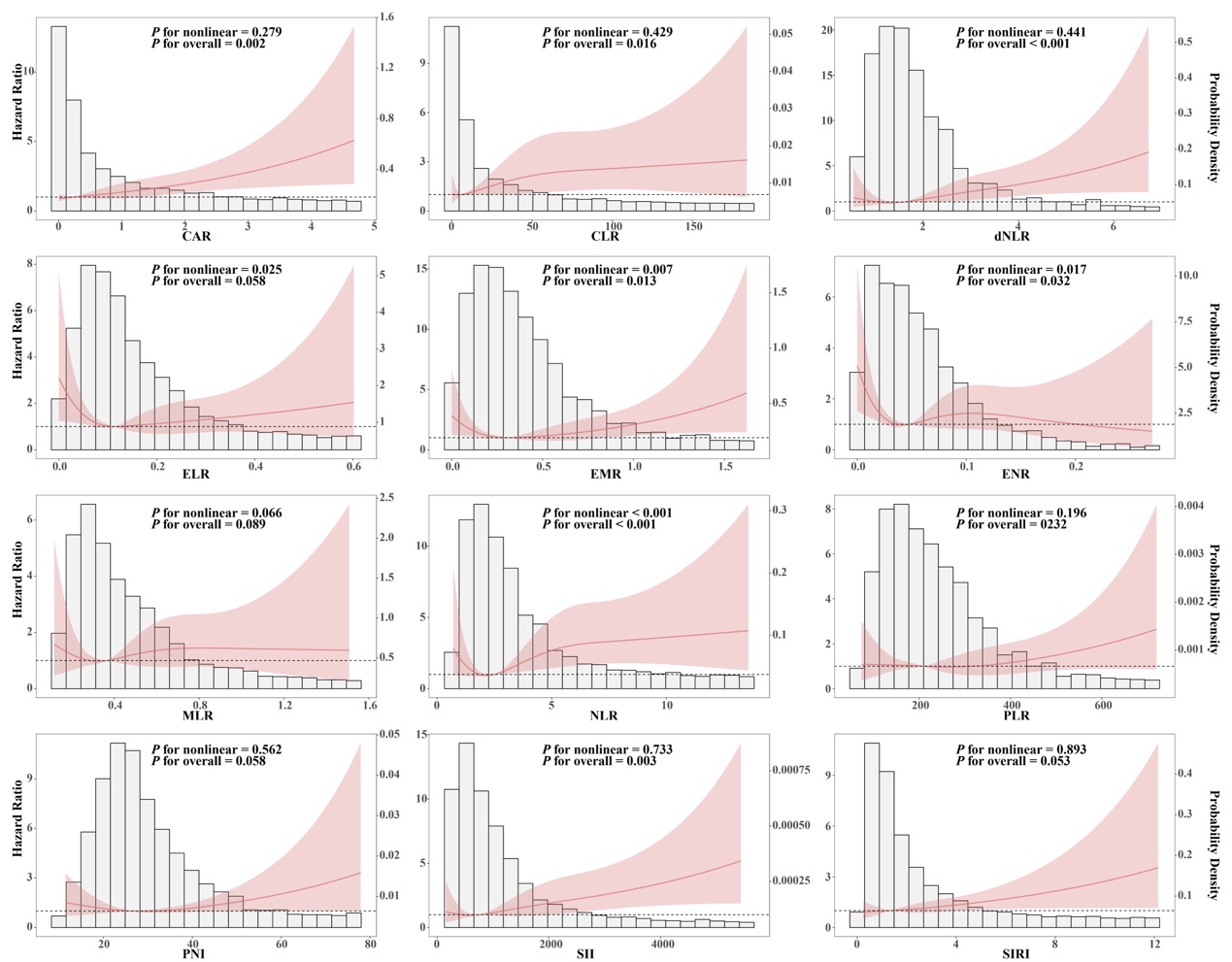


Supplementary Figure 4 RCS analysis between inflammatory indices and unfavorable treatment outcomes among patients without comorbidities. The red line and shaded area represent the Hazard Ratio and its 95% confidence interval, while the bars represent the probability density. (A) CAR: C-reactive protein to albumin ratio; (B) CLR: C-reactive protein to lymphocyte ratio; (C) dNLR: Derived neutrophil to lymphocyte ratio; (D) ELR: Eosinophil to lymphocyte ratio; (E) EMR: Eosinophil to monocyte ratio; (F) ENR: Eosinophil to neutrophil ratio; (G) MLR: Monocyte to lymphocyte ratio; (H) NLR: Neutrophil to lymphocyte ratio; (I) PLR: Platelet to lymphocyte ratio; (J) PNI: Prognostic Nutritional Index; (K) SII: Systemic Immune-Inflammation Index; (L) SIRI: Systemic Inflammatory Response Index.


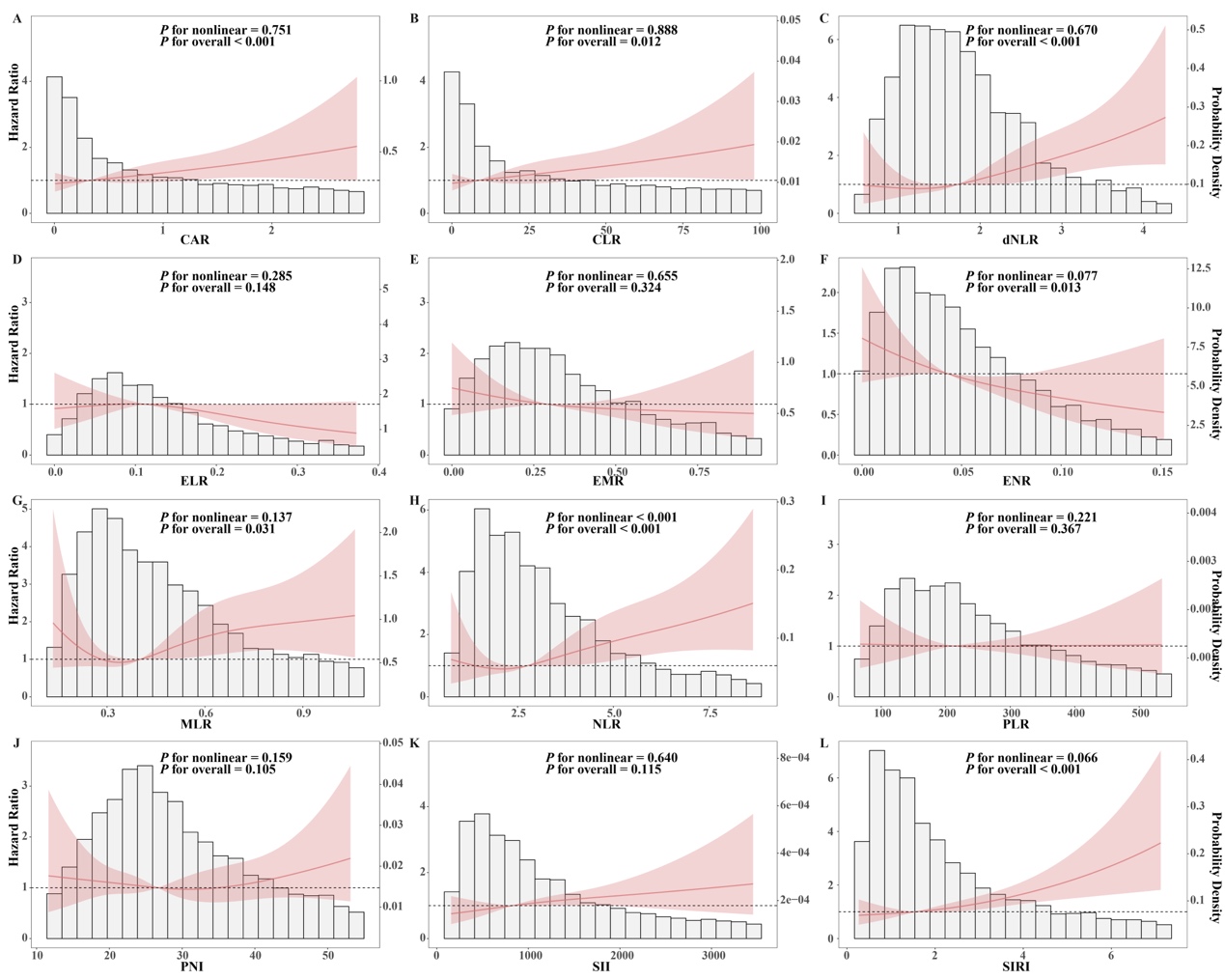


Supplementary Figure 5 RCS analysis between inflammatory indices and unfavorable treatment outcomes among patients with complete data. The red line and shaded area represent the Hazard Ratio and its 95% confidence interval, while the bars represent the probability density. (A) CAR: C-reactive protein to albumin ratio; (B) CLR: C-reactive protein to lymphocyte ratio; (C) dNLR: Derived neutrophil to lymphocyte ratio; (D) ELR: Eosinophil to lymphocyte ratio; (E) EMR: Eosinophil to monocyte ratio; (F) ENR: Eosinophil to neutrophil ratio; (G) MLR: Monocyte to lymphocyte ratio; (H) NLR: Neutrophil to lymphocyte ratio; (I) PLR: Platelet to lymphocyte ratio; (J) PNI: Prognostic Nutritional Index; (K) SII: Systemic Immune-Inflammation Index; (L) SIRI: Systemic Inflammatory Response Index.


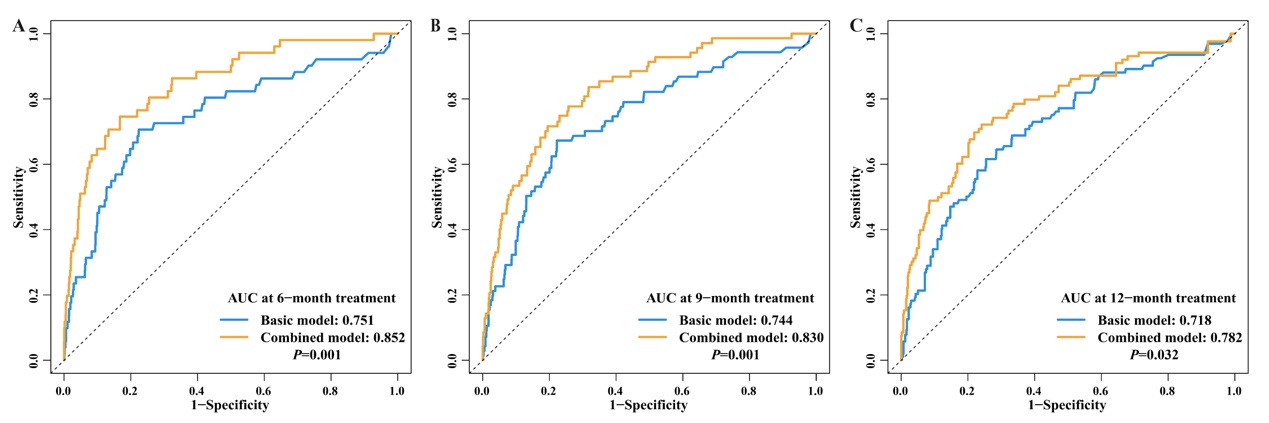


Supplementary Figure 6 Predictive value for treatment outcome among patients without comorbidities between basic model and combined model. (A) AUC at 6-month treatment; (B) AUC at 9-month treatment; (C) AUC at 12-month treatment Note: Basic model: included age, sex, BMI, work status, drinking, smoking, treatment category, cavity, and DST. Combined model: a combination of blood inflammatory indices, added to the characteristics included in the basic model. BMI: Body Mass Index; DST: Drug susceptibility testing; AUC: Area under curve.


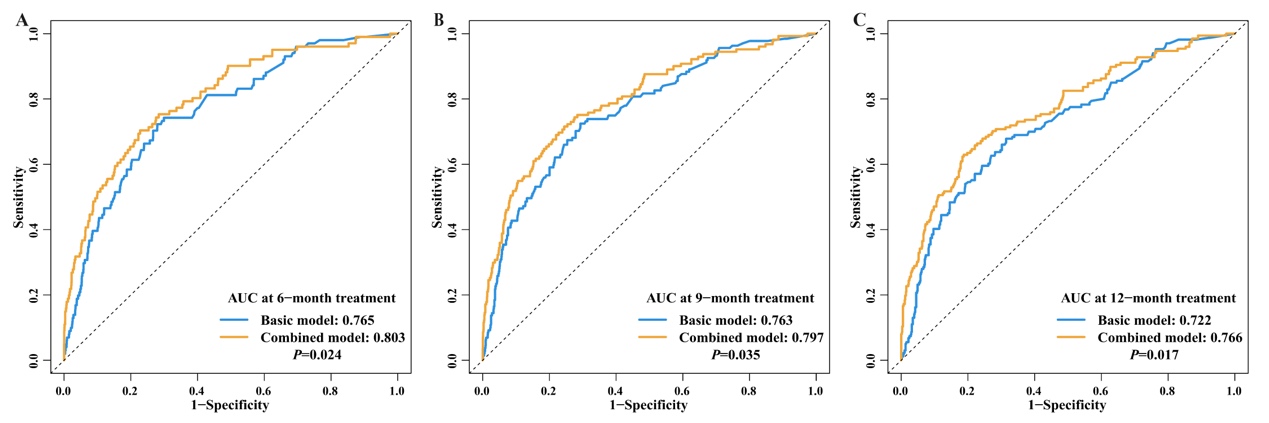


Supplementary Figure 7 Predictive value for treatment outcome among patients with complete data between basic model and combined model. (A) AUC at 6-month treatment; (B) AUC at 9-month treatment; (C) AUC at 12-month treatment Note: Basic model: included age, sex, BMI, work status, smoking, treatment category, fatigue, weight loss, diabetes, hypertension, tumor, anemia, cavity, DST, and MTB2. Combined model: a combination of blood inflammatory indices, added to the characteristics included in the basic model. BMI: Body Mass Index; DST: Drug susceptibility testing; MTB2: Bacteriological test result at 2-month treatment; AUC: Area under curve.

Reference：

1. Linh N N, Viney K, Gegia M, Falzon D, Glaziou P, Floyd K, et al. World health organization treatment outcome definitions for tuberculosis: 2021 update[J]. European Respiratory Journal, 2021, 58(2): 2100804. DOI:10.1183/13993003.00804-2021.
